# Assessing Patient-Centred Outcomes in Lateral Elbow Tendinopathy: A Systematic Review and Standardised Comparison of English Language Clinical Rating Systems

**DOI:** 10.1186/s40798-019-0183-2

**Published:** 2019-03-20

**Authors:** Jonathan Peter Evans, Ian Porter, Jaheeda B. Gangannagaripalli, Charlotte Bramwell, Antoinette Davey, Chris D. Smith, Nicola Fine, Victoria A Goodwin, Jose M Valderas

**Affiliations:** 1 Health Services and Policy Research Group, University of Exeter Medical School, Exeter, UK; 2 Royal Devon and Exeter NHS Foundation Trust, Exeter, UK; 3 National Institute for Health Research (NIHR) Collaboration for Leadership in Applied Health Research and Care (CLAHRC) South West Peninsula, University of Exeter Medical School, Exeter, UK

**Keywords:** Tennis elbow, Lateral elbow tendinopathy, Patient-reported outcome measures, PROMs, Psychometrics, Validation

## Abstract

**Background:**

Lateral elbow tendinopathy (LET) is a common condition affecting adults. Although a lack of treatment consensus continues to prompt numerous effectiveness studies, there is a paucity of clear guidance on the choice of outcome measure. Our aim was to undertake a standardised evaluation of the available clinical rating systems that report patient-centred outcomes in LET.

**Methods:**

A systematic review of studies reporting the development, assessment of metric properties and/or use of instruments aiming to quantify LET-specific patient-centred outcome measures was conducted in MEDLINE, Embase and CINAHL (inception-2017) adhering to PRISMA guidance. The evidence for each instrument was independently assessed by two reviewers using the standardised evaluating measures of patient-reported outcomes (EMPRO) method evaluating overall and attribute-specific instrument performance (metric properties and usability). EMPRO scores > 50/100 were considered indicative of high performance.

**Results:**

Out of 7261 references, we identified 105 articles reporting on 15 instruments for EMPRO analysis. Median performance score was 41.6 (range 21.6–72.5), with four instruments meeting high-performance criteria: quick Disabilities of the Arm Shoulder and Hand score (qDASH) (72.5), DASH (66.9), Oxford Elbow Score (OES) (66.6) and Patient-Rated Tennis Elbow Evaluation (PRTEE) (57.0). One hundred seventy-nine articles reported instrument use internationally with DASH as the most frequent (29.7% articles) followed by PRTEE (25.6%), MEPS (15.1%) and qDASH (8.1%). The correlation between frequency of use and performance was *r* = 0.35 (95%CI − 0.11; 0.83).

**Conclusions:**

This is the first study to provide standardised guidance on the choice of measures for LET. A large number of clinical rating systems are both available and being used for patients with LETs. Robust evidence is available for four measures, the DASH, QDASH, PRTEE and OES. The use of instruments in the literature is only in part explained by instrument performance.

**Electronic supplementary material:**

The online version of this article (10.1186/s40798-019-0183-2) contains supplementary material, which is available to authorized users.

## Key Points

- There are 15 clinical rating systems used in the literature that aims to quantify the patients’ experience of lateral elbow tendinopathy. Adequate evidence of their validity is only available for four of those 15 clinical ratings systems.
- Within the literature, the choice of the clinical rating system has not been associated with its quality.

## Background

Lateral elbow tendinopathy (LET), known commonly as tennis elbow, is a highly prevalent and painful condition affecting the lateral side of the elbow [1]. The exact aetiology and pathogenesis are currently unknown and are the source of much debate [2–7], but some element of overuse of the common forearm extensors is believed to be implicated. Patients experience a broad spectrum of symptoms, from mild self-limiting pain that responds to activity modification and simple analgesia, to debilitating pain on the outer elbow, spreading down the forearm, which leads to loss of function of the affected limb [1]. At its worst, LET can have a major impact on an individual’s social and professional life [8, 9].

The general population prevalence of LET is between 1% and 3% [10], rising in those with occupational risk factors to as high as 41% [11]. Men and women are equally affected and onset peaks at approximately 40–50 years [12, 13]. Although this condition is usually self-limiting, symptoms persist for over 1 year in up to 20% of people, and in 8.5%, symptoms recur within 2 years [14]. Up to 5% of LET patients claim sickness absence, at an average duration of 29 days per year [15]. In the UK in 2012, lost earnings through absenteeism from LET was estimated to cost £27 million [16].

There is no consensus on the optimal management of LET [17]. Though a vast quantity of options exists, large systematic evaluations continue to find deficits in the evidence base for many interventions [10]. Recommendations for future research include conducting large-scale, good quality randomised controlled trials (RCTs) that utilise validated outcome measures [1, 10, 13, 18]. Historically, outcomes in LET focused on clinical examination findings; however, recent emphasis in health technology assessment has shifted to focus on quantifying the patients’ perspective on how their condition affects their life.

In an effort to capture the effect of health interventions on patients, there has been a considerable investment of resources by academics and clinicians to develop standardised, robust and valid ways of collecting patient-centred outcomes, typically collected through clinical rating systems that are either patient-reported outcome measures (PROMs) or clinician-reported outcomes measures (CROMs), sometimes also combining both approaches [19]. Their choice and use must be supported by published evidence demonstrating that they are acceptable to patients, reliable, valid and responsive (sensitive to change) [20]. Furthermore, in an effort to bring further standardisation across research and clinical applications, clinical rating systems are also being integrated into core outcome sets, with influential groups including the Core Outcome Measures in Effectiveness Trials (COMET) and International Consortium for Health Outcomes Measurement (ICHOM) placing particular emphasis on a systematic approach to instrument choice.

The 2013 review by The et al. [21] represents the only previous attempt at a standardised assessment of elbow specific measures. They included 12 outcome measures using the Consensus-Based Standards for the Selection of health Measurement Instruments (COSMIN) checklist which concluded that the Oxford Elbow Score (OES) was developed using the highest quality methodology. However, for certainty of use, the performance of clinical rating systems needs to be assessed specifically for the condition of interest. As The et al. comment, it is essential to be aware that established validity characteristics might not be applicable when using the rating system in a different population. Therein, a rating system originally designed for the assessment of elbow joint arthroplasty may not be appropriate for use in LET, unless evidence of performance has been explicitly presented. Furthermore, a narrow focus on elbow specific instruments neglects the possibility that region-specific (i.e. upper limb) instruments may have been robustly assessed for certain pathologies. Finally, reviews of measures with a region/anatomical location perspective disregard the very different characteristic clinical presentations of different conditions and may advise on the use of measures that works best across conditions, but not necessarily the best option for any given condition.

To our knowledge, no systematic review has previously identified the clinical rating systems for patients with LET and assessed both their use in the scientific literature and their performance against validated standards, both to establish what instruments offer the best performance and whether these are indeed the ones most widely used. This study aims to apply a standardised system to evaluate evidence available on the metric properties, development process and utility of clinical rating systems assessing patient-centred outcomes in LET.

## Methods

### Systematic Review

We conducted a systematic review of published studies on the development and/or use of clinical rating systems in patients with LET. This systematic review was registered with the PROSPERO International prospective register of systematic reviews (PROSPERO 2016:CRD42016037317), and the present report has been written following PRISMA guidelines [22].

A search strategy was constructed using MeSH and free-text terms (Additional file 1) (available at http://medicine.exeter.ac.uk/research/healthserv/healthservicesandpolicy/projects/proms/optimisinginterventionaltreatmentoftenniselbow/elbowoutcomemeasures/#d.en.504281).

The search strategy development was guided by previously published strategies for systematic reviews of interventions in LET [23] and for the identification of outcome measures [24]. Known condition-specific measures were identified and incorporated into the strategy through the assessment of previous systematic reviews of elbow-specific rating scales [21, 25–27], and search of the online library of patient-reported outcomes and quality of life database (PROQOLID) [28].

The strategy was tailored to each database through the modification of thesaurus terms, wildcards and truncations. The search was first run on 1 May 2017 in Medline (Ovid MEDLINE, 1948 to 2017 & Ovid MEDLINE In-Process & Non-Indexed Citations) accessed through OVIDSP, Embase (Embase 1974 to 2017) accessed through OVIDSP and CINAHL (CINAHL 1981 to 2017). Further searches of the Cochrane Central Register of Controlled Trials (CENTRAL), thesis searching via ProQuest and the Kings Fund library database were undertaken.

The resulting references were retrieved and imported into a bibliographic database using reference manager software (Endnote X7). Duplicates were removed.

All articles reporting the development, psychometric evaluation or use of English language clinical rating systems in LET in adults (> 18 years) were included. In instances where the study included multiple elbow pathologies, it had to specify that this comprised, at least in part, a population of LET patients. Multi-item upper limb or elbow-specific instruments that were either clinician or patient-led were included.

Study selection utilised a step-wise approach. Screening was conducted by two reviewers at all stages. To ensure the highest levels of sensitivity, in cases of disagreement, the study proceeded to the next step for more in-depth assessment. Reviewer comprehension of the research aims was assessed using a 20-study pilot, achieving an inter-rater agreement (kappa) of 0.85. Title and abstracts were disseminated to the reviewers using the web and mobile application software Rayyan (Doha, Qatar) which allows collaborators to remotely screen the articles [29]. Full-text assessment was undertaken using hard copy manuscripts. Studies were excluded if reporting case studies, case reports, surgical technique papers, conference abstracts and manuscripts not in the English language. Forward and backward searches were undertaken on full-text manuscripts using Scopus® (Elsevier B.V.). Instrument manuals, complementary support material and cross-check of reference lists were sourced via the instruments’ associated website or in direct contact with the developer.

Due to the principles of cross-cultural adaptation, the metric properties of an instrument are not directly comparable across different versions. Hence, only full texts of instruments developed or tested in the English speaking populations were included in the evaluating measures of patient-reported outcomes assessment (EMPRO) [30]. By convention, the instruments were identified by their name and acronym, when one had been given or by the name of the first author in the seminal paper, and the clinical rating systems were classified as either PROMs, pure CROMs and mixed PROMs/CROMs.

### Evaluating Measures of Patient-Reported Outcomes (EMPRO)

The EMPRO tool [30] was developed to measure the performance of patient-centred outcomes for informing the identification of the best candidates among measures competing for the same purpose. Originally designed for PROMs, the content, structure and methodology are apt for the evaluation of all clinical rating systems. It has been utilised in a number of areas, including assessment of shoulder outcome instruments [31] and there is good evidence for its validity and reliability [30]. Its particular strength includes the synthesis of the whole body of evidence surrounding an outcome instrument and its ability to facilitate the selection of the most appropriate outcome instrument [30]. Unlike the COSMIN checklist, it does not evaluate the quality and design of the evaluation of the psychometric properties but rather the performance of the instrument.

EMPRO consists of eight scales measuring the following attributes each: conceptual and measurement model (7 items), reliability (8), validity (6), responsiveness (3), interpretability (3), administrative burden (7), alternative modes of administration (2) and cross-cultural adaptations into chosen reference language (3). Each item consists of a short statement, together with suggested aspects to be considered. Reviewers then express their agreement on an ordinal Likert-type response scale of 1–4. Where appropriate, ‘not applicable’ and ‘no information available’ response categories are available. At the end of the tool, reviewers are requested to provide an overall recommendation [30] (Table 1).

**Table 1:** EMPRO attributes definition, number of items and scoring description (adapted from Garin et al. [36]). *KR-20* Kuder-Richardson 20, *EMPRO* evaluating measures of patient-reported outcomes

Attribute	Definition	No. of items	Higher scores represent…
Conceptual and measurement model	The rationale for and description of the concept and the populations that a measure is intended to assess and the relationship between these concepts	7	The concept is more clearly stated to be measured. The empirical basis and methods for obtaining the item and for combining them are more appropriate
Reliability	The degree to which an instrument is free from random error	8	More clearly described and superior methods to collect internal consistency data. Better values of Cronbach’s alpha and/or KR-20 coefficients
Validity	The degree to which the instrument measures what it purports to measure	6	More evidence regarding content-related validity of the instrument for its intended use
Responsiveness	An instrument’s ability to detect change over time	3	More clearly described and more appropriate methods to assess sensitivity to change. The estimated magnitude of change is more clearly described, and the results are better
Interpretability	Possibility of assigning meaning to quantitative scores	3	The strategies to facilitate interpretation are more clearly described and appropriate
Burden	The time, effort and other demands placed on those to whom the instrument is administered (respondent burden) or on those who administer the instrument (administrative burden)	7	The skills and time to complete the instrument are more clearly described and acceptable
Alternative modes of administration	Alternative modes of administration used for the administration of the instrument	2	The metric characteristics and use of each alternative mode of administration are specifically described and adequate

Each instrument was evaluated independently by two researchers using the EMPRO tool and based on the following information:

- The instrument to be assessed
- The instrument’s user manual (where available)
- Full text of all publications which provide information concerning the development process, the metric properties or the administration of the instrument including a sample which, at least in part, contains participants with LET.

The researchers were experts in outcomes research, they received additional training in the use of the EMPRO, and none of them had been involved in the development of the reviewed measures. EMPRO scores were consolidated and tabulated. Where discrepancy in scores existed, the two reviewers initially discussed the case to resolve through consensus, where necessary a third reviewer opinion was sought.

### Analytic Strategy

Attribute specific scores were calculated as the response mean of the applicable items. Items for which the response was ‘no information’ were assigned a score of 1 (lowest possible). This raw mean was linearly transformed to scale the scores from 0 (worst possible) to 100 (best possible).

From the attribute scores, an overall attribute mean score was calculated. The scores of the five attributes that relay the psychometric-related information (conceptual and measurement model, reliability, validity, sensitivity to change and interpretability) were included. The overall attribute score was only calculated when at least three of the five attributes had a score. EMPRO overall attribute scores for each outcome instrument are considered adequate if they reach at least 50 out of the maximum score of 100 [30].

Agreement between reviewers was assessed using a weighted Cohen’s kappa coefficient. All analysis was undertaken in STATA (2015. Release 14. College Station, TX: StataCorp LP). Databases of instruments’ distribution were managed in MS Excel (2013, Redmond, WA: Microsoft®). Kappa scores and resource numbers are displayed as median (interquartile range (IQR)) range). Spearman’s correlation coefficient was used to assess the relationship between EMPRO score and proportional use of the instrument within the literature.

## Results

**Fig. 1:**
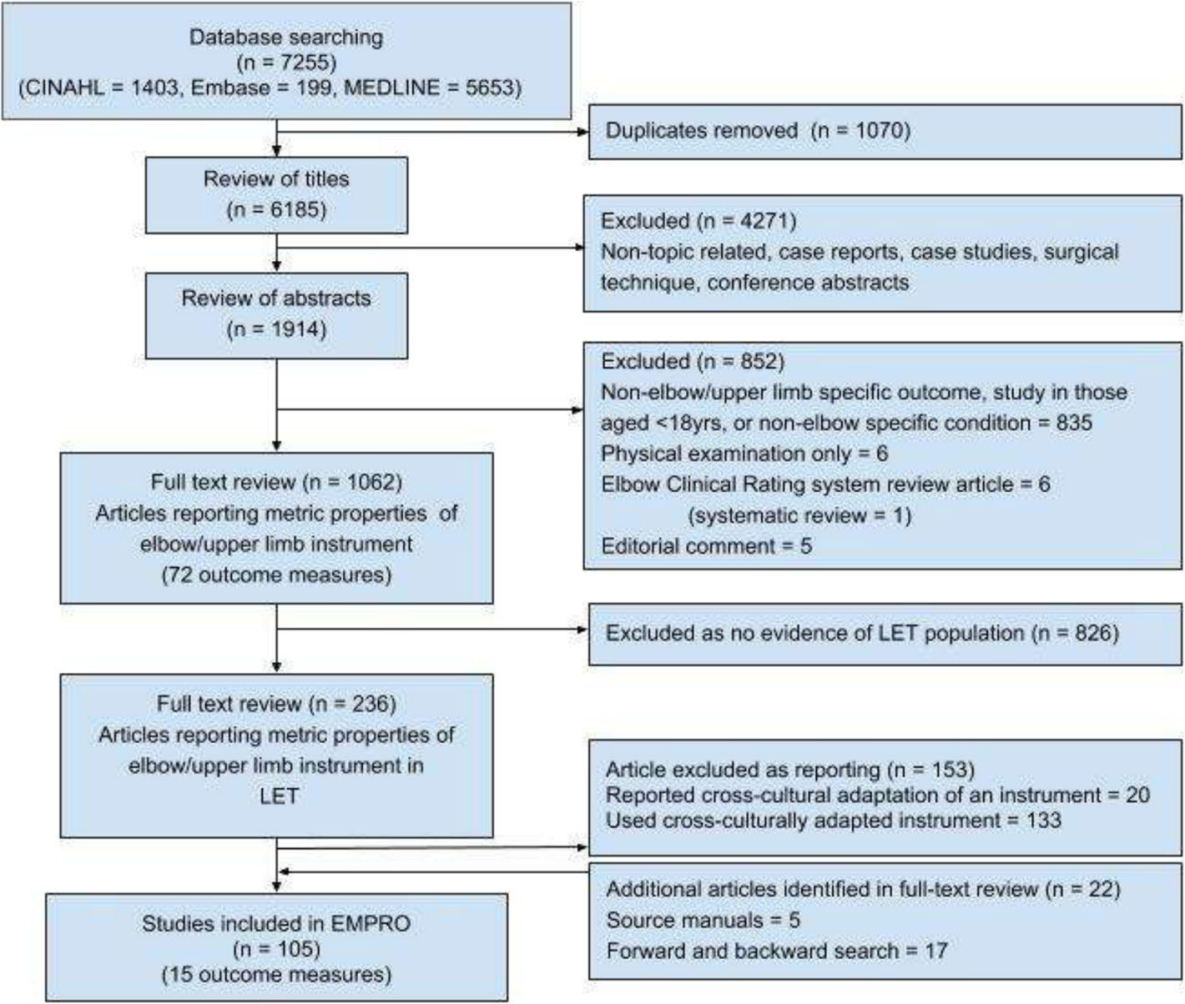
PRISMA flowchart of systematic literature review. Review of articles reporting development/metric properties or use of outcome instruments. LET lateral elbow tendinopathy, EMPRO evaluating measures of patient-reported outcomes

The review search strategy identified 7261 articles (Fig. 1). Following duplicate removal, 6185 articles were reviewed at the title level. After evaluation of references screened as full texts, 15 clinical rating scales were identified (Table 2).

**Table 2:** Summarised characteristics of the 15 identified outcome instruments. *LET* lateral elbow tendinopathy, *VAS* visual analogue scale

Instrument	Author (year)	Development purpose	Assessor	Dimensions (no. items)	Scales	No. documents on development and metric properties (see Additional file 2)	No. documents reporting instrument use internationally (English speaking) (see Additional file 2)	Data from an exclusively LET population or mixed pathology
A&C (Andrews and Carson)	Andrews et al. [45]	To evaluate subjective and objective results of elbow arthroscopy	Clinician	Symptoms (3) Activities (1) Function (3)	4-point Likert scale scored out of 200 then interpreted as one of four groups (poor–excellent)	1	2 (2)	Mixed
ASES-E (American Shoulder and Elbow Score-E)	King et al. [46]	Elbow functional assessment	Patient and clinician	Pain (5) Function (12) Satisfaction (1) + Clinical assessment	Mixture of visual analogue scale and 4-point Likert scales.	3	5 (5)	Mixed
DASH (Disabilities of the Arm Shoulder and Hand)	Hudak et al. [47]	Region (arm) specific measure of disability and symptoms with any or multiple musculoskeletal disorders of the upper limb.	Patient	Physical function (21) Symptoms (5) Psychosocial (4) (optional work and sport/music module)	5-point Likert scale Raw score converted to 0–100 scale	18	60 (23)	Mixed
HSS (Hospital for Special Surgery)	Inglis and Pellicci [48]	Pre and post of assessment of elbow arthroplasty	Clinician	Pain (2) Function (2) + Clinical assessment	Categorical scoring of pain at rest (5 options) and in bending (4). Function split into A (4) and B (5) + clinical assessment Scored 0–100	3	1 (1)	Mixed
LES (Liverpool Elbow Score)	Sathyamoorthy et al. [49]	Elbow specific measure of function and clinical state	Patient and clinician	Physical function (8) Pain (1) + Clinical assessment	5-point Likert scale Raw score converted to 0–10 scale	1	1 (0)	Mixed
MEPS (Mayo Elbow Performance Score)	Morrey and Adams [50]	For the assessment of total elbow arthroplasty	Clinician	Pain (5) Function (15) + Clinical assessment	10-point Likert scale Scored out of 100 then interpreted as one of four groups (poor–excellent)	6	24 (9)	Mixed
Morrey	Broberg and Morrey [51]	For the assessment of radial head fractures excision	Clinician	Pain (1) + Clinical assessment	Categorical scoring of pain (4 options) Scored out of 100 then interpreted as one of four groups (poor–excellent)	2	4 (0)	Mixed
Nirschl	Nirschl [52]	Assessment of LET based on phases of pain.	Patient and clinician	Pain (1) + Addition of VAS and surgical findings	Categorical scoring of pain (7 options)	2	16 (7)	LET
OES (Oxford Elbow Score)	Dawson et al. [53]	For the assessment of the outcome of elbow surgery	Patient	Pain (4) Function (4) Limitation to work and leisure activities (2) Psychosocial (2)	Categorical scoring options Converted to numerical value (0–4) Domains scored individually	5	5 (2)	Mixed
PRTEE (Patient-Rated Tennis Elbow Evaluation) (formally PRFE)	Overend et al. [54]	For measurement of forearm pain and disability in patients with LET	Patient	Pain (5) Function • Specific (6) • Usual (4)	10-point Likert scale Raw score converted to 0–100 scale	9	53 (21)	LET
qDASH (quick Disabilities of the Arm Shoulder and Hand)	Beaton et al. [55]	Abbreviated DASH score	Patient	Physical function (6) Pain (2) Psychosocial (3)	5 point Likert scale Raw score converted to 0–100 scale	8	18 (5)	Mixed
R&M (Roles and Maudsley)	Roles and Maudsley [56]	To classify the outcome of surgery in radial tunnel syndrome	Clinician	Pain Movement Activity	Placed in 1 of 4 groups (poor–excellent) dependent on composite of dimension finding	2	16 (2)	Mixed
TEFS (Tennis Elbow Functional Score)	Lowe [39]	For the assessment of disability in patients with LET	Patient	Pain (10)	5-point Likert scale Scores of 10 items added together	1	3 (0)	LET
ULFI (Upper Limb Functional Index)	Pransky et al. [57]	For the assessment of upper limb function	Patient	Function (8)	10-point Likert scale Scores of 8 items added together	1	6 (3)	Mixed
Verhaar	Verhaar et al. [58]	For the assessment of the outcome of surgery in LET	Clinician	Pain Satisfaction Movement Strength	Placed in 1 of 4 groups (poor–excellent) dependent on composite of dimension finding	1	6 (1)	LET

**Fig. 2:**
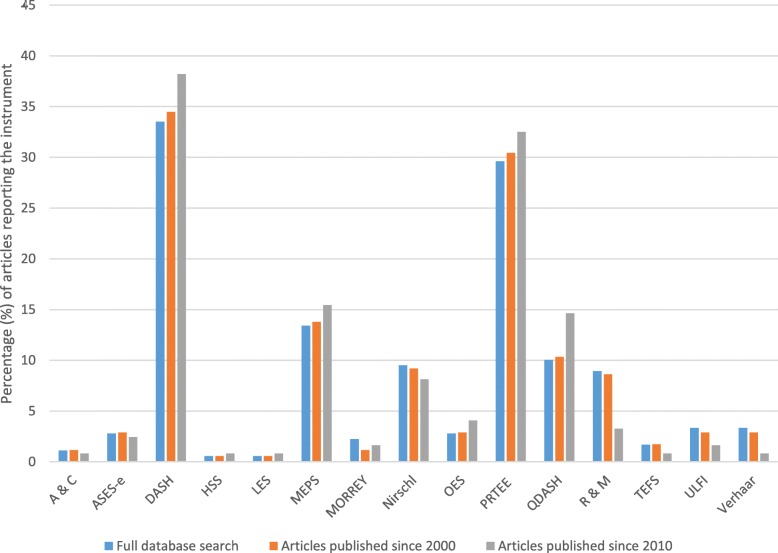
The change in the percentage of use of outcome instruments over time. A&C Andrews and Carson, ASES-E American Shoulder and Elbow Score-E, DASH Disabilities of the Arm Shoulder and Hand, HSS Hospital for Special Surgery, LES Liverpool Elbow Score, MEPS Mayo Elbow Performance Score, OES Oxford Elbow Score, PRTEE Patient-Rated Tennis Elbow Evaluation, qDASH quick Disabilities of the Arm Shoulder and Hand, R&M Roles and Maudsley, TEFS Tennis Elbow Functional Score, ULFI Upper Limb Functional Index

Assessment of the instruments’ reported use in LET studies found four instruments to be reported much more frequently than the remaining 11 (Fig. 2). The Disabilities of the Arm Shoulder and Hand (DASH) score was the most frequently reported (29.7% of articles), followed by the Patient-Rated Tennis Elbow Evaluation (PRTEE) (25.6%), Mayo Elbow Performance Score (15.1%) and quick Disabilities of Arm Shoulder and Hand (qDASH) (8.1%). Over time, this trend has shifted with the reporting of these scores increasing over time. Of note, of the 179 articles in the international literature, 40 (22.3%) reported two using two or more clinical rating systems to assess patient-centred outcomes. Within the 179 articles, 155 reported the results of clinical effectiveness research, 36 (23.2%) of which were surgical, 117 (75.5%) were non-surgical and 2 (1.3%) compared surgical and nonsurgical modalities. Differences were noted in the proportional use of the most common outcome measures (DASH, PRTEE, MEPS and qDASH) within the surgical (41.7%, 8.3%, 25% and 5.6%, respectively) and non-surgical group (28%, 23%, 7.7% and 7.7%).

### Clinical Rating Systems

Of the 15 outcome clinical rating systems, six were PROMs, six were CROMs, and the remaining three included both clinician and patient-reported information (Table 2).

The instruments had been developed between 1979 and 2008. Four instruments had been designed specifically for the assessment of LET: Patient-Rated Tennis Elbow Evaluation (PRTEE), Nirschl score, Tennis Elbow Functional Scale (TEFS) and Verhaar score. Three more instruments had been designed as elbow-specific across different pathologies: American Society of Shoulder and Elbow Surgeons-Elbow (ASES-E), Liverpool Elbow Score (LES) and Oxford Elbow Score (OES). Three other instruments (DASH, qDASH and the Upper Limb Functional Index (ULFI)) are region-specific (upper limb), and the remaining five instruments had been designed for the assessment of other pathologies (e.g. arthroplasty, radial head fracture) but have been used in the assessment of LET outcomes.

### Psychometric Evaluation

All instruments were assessed using the EMPRO methodology (Additional file 2). The volume of resources informing each EMPRO assessment averaged four articles (IQR 8.5) (range 1–41) (Table 2).

**Fig. 3:**
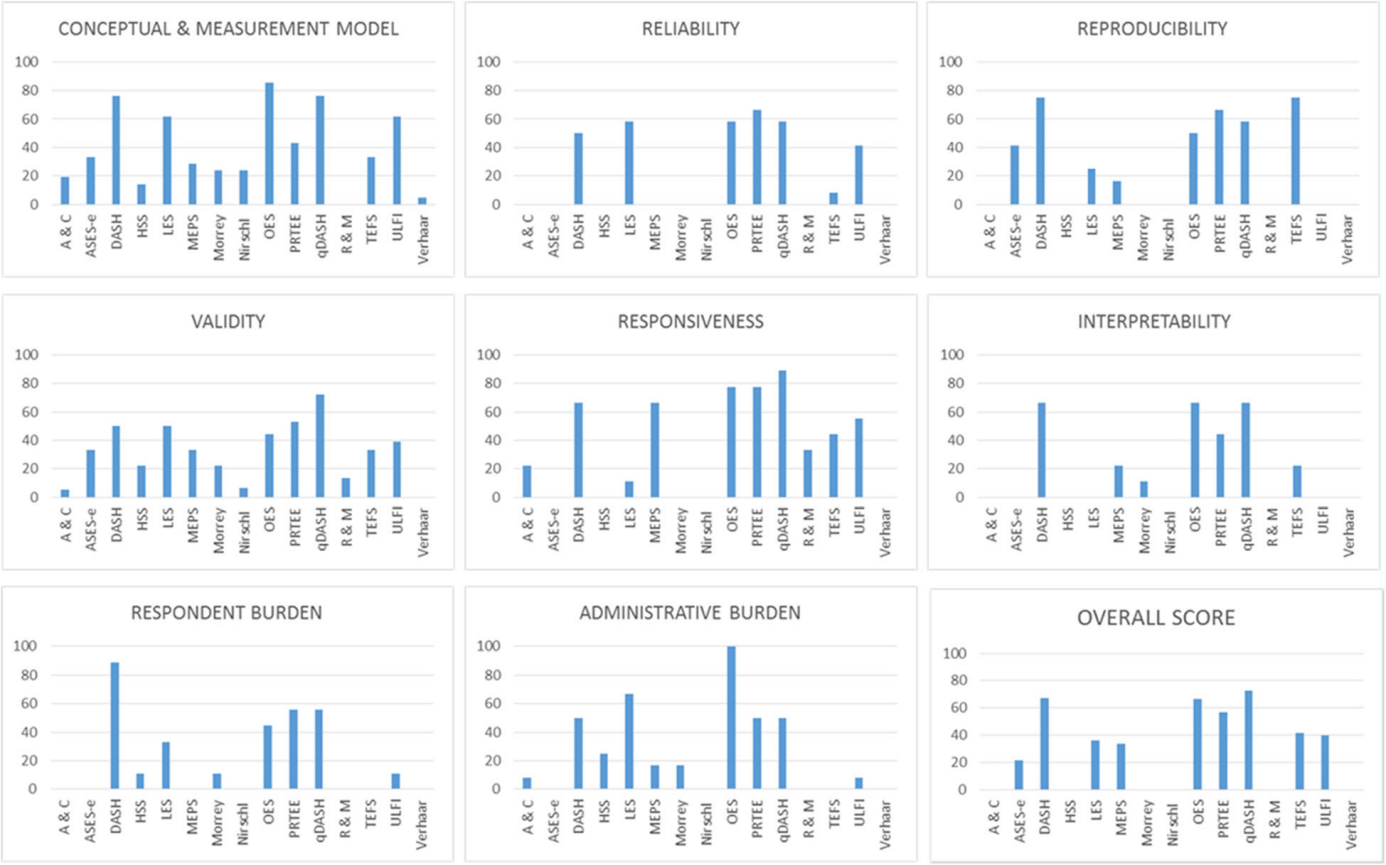
Attribute specific and overall EMPRO scores. 0 (worst) to 100 (best). A&C Andrews and Carson, ASES-E American Shoulder and Elbow Score-E, DASH Disabilities of the Arm Shoulder and Hand, HSS Hospital for Special Surgery, LES Liverpool Elbow Score, MEPS Mayo Elbow Performance Score, OES Oxford Elbow Score, PRTEE Patient-Rated Tennis Elbow Evaluation, qDASH quick Disabilities of the Arm Shoulder and Hand, R&M Roles and Maudsley, TEFS Tennis Elbow Functional Score, ULFI Upper Limb Functional Index, EMPRO evaluating measures of patient-reported outcomes

Concordance between individual EMPRO evaluations was moderate to substantial in all cases, kappa median 0.72 (IQR 0.36) (range 0.47–0.94) [32]. Resolution of score differences was achieved by consensus in all cases. The overall summary scores ranged from 72.5 (qDASH) to 21.6 (ASES-E). Only four instruments met the threshold score of 50/100: one LET specific (PRTEE), one elbow specific (OES) and two upper-limb specific (qDASH and DASH). It was not possible to calculate the overall scores for the Morrey, Andrews and Carson, Roles and Maudsley, Hospital for Special Surgery score (HSS), Nirschl and Verhaar instruments because of a lack of available evidence (Fig. 3).

Whereas no reviewer ‘strongly recommended’ any of the outcome instruments, qDASH, DASH, OES and PTREE were all ‘recommended (with provisos or alterations)’. Of those instruments, recommendations of use extended only to group comparison of a general adult population rather than individual monitoring, owing to lack of clear responsiveness data in LET patients.

The Spearman correlation coefficient between overall performance and frequency of use in the literature was *r* = 0.35 (95%CI − 0.11; 0.83).

## Discussion

This study identified 15 clinical rating systems that, to varying degrees, attempt to assess patient outcomes in individuals suffering from LET. All 15 clinical rating systems were systematically evaluated in view of their development, metric properties and history of use within the LET literature. Of those instruments, only four met both the overall attribute benchmark score of 50 and overall recommendation of the reviewers, to suggest that their use can be justified in the evaluation of LET. This study has gone a step further than previous elbow-specific outcome instrument evaluations [21, 25, 33–35] in attempting to systematically compare the instruments in a condition-specific context. Furthermore, it is the first to attempt to quantify both the properties of the instruments and the instruments’ distribution of use within the literature, which both feature significantly in the researcher’s or clinician’s mind when choosing a tool. From this assessment, we would recommend authors of future studies of LET participants, where English language instruments will be used, consider the qDASH, DASH, OES or PRTEE. Furthermore, summary tables from the EMPRO evaluation (Additional file 3) can be used to guide instrument choice when the quantification of a particular attribute is desirable. For example, if the responsiveness in longitudinal studies is a priority, we would recommend the qDASH or PRTEE; for minimised administrative burden, we would recommend the OES; if the inclusion of specific dimensions such as psychosocial effect was desired, we would recommend the OES. This presentation of condition-specific quality may also reduce the 22% of studies that utilise two or more clinical rating systems, with its consequent burden on the study participants.

To the authors’ knowledge, this is the first upper-limb specific study that has quantified the condition-specific quality of the instrument, and formally identified instruments’ distribution of use. Previous EMPRO evaluations have found concordance between the quality of the instruments and their history of use [36]. Though there is some agreement between quality and use of instruments in LET, instruments are being widely used although the evidence for their metric properties is significantly limited compared to alternatives. Whilst the results of our standardised evaluation would support the common use of the DASH and PRTEE, it is surprising that the qDASH and OES are used so infrequent. Furthermore, it is concerning that the Mayo Elbow Performance Score (MEPS), which did not meet our minimum benchmark, is used twice as often as the qDASH and seven times more often than the OES. This trend, which has not changed significantly over time, would appear to be more prevalent within the surgical rather than non-surgical literature. The qDASH is the abbreviated version of the DASH and scored more highly than the full version owing to a more compelling record of validation in LET populations. Although it is becoming increasingly popular, it is still only utilised in a minority (14.6%) of contemporary LET studies.

### High-Performing Instruments

The qDASH, DASH, OES and PTREE exceeded the minimum criteria for recommendation. Of note, both the qDASH and DASH scored > 50 on every attribute-specific score. Of these four measures, particular strengths (identified as a particular metric attribute-specific score > 80/100), were the conceptual development of the OES and responsiveness of the qDASH. The OES developed its items with patient groups and expert panels, using a high-quality methodology; however, it is worth noting that due to the unidimensional nature of the resulting instrument, composite scores are not advised, a fact ignored in many subsequent studies using the score. The responsiveness of the qDASH has been complemented by studies containing considerable proportions of LET patients [37, 38]; however, it is worth noting that the DASH, OES and PRTEE all scored well in this attribute. The condition-specific PRTEE, though reliable, valid and responsive, was developed without clear patient involvement. Factor analysis, where the number of fundamental dimensions that underlie the observed data is analysed statistically in a large dataset, in order to rationalise the questionnaire structure has not been reported, and the justification of visual numeric scales is not clear.

Areas where further data could enhance these scores include assessment of metric properties in isolated LET groups for the qDASH, DASH and particularly the further assessment of condition-specific construct validity of the OES. Furthermore, future studies focusing on the interpretability would benefit from a LET-specific derivation of minimal change scores through the use of either accepted distribution-based or anchor-based methodologies, which would strengthen this specific attribute considerably.

### Other Instruments

The TEFS, ULFI, LES, MEPS and ASES-E scored below the minimum criteria for recommendation. Though the TEFS is a condition-specific score, the reporting of its metric properties has only occurred in a University Master’s thesis published in 1999 [39]. However, it has a history of use in peer-reviewed publications as recently as 2012. Though scoring well for reproducibility, the weight of evidence for the remaining metric properties currently precludes its recommendation. The ULFI is a generic upper limb score with a history of use in LET, and although conceptually well designed and responsive, the lack of information on its metric properties within a condition-specific context precludes its recommendation. The LES is a robustly designed instrument that has been employed in LET studies; however, a significant lack of data on the instrument’s responsiveness and interpretability hugely hamper the instrument’s utility to the researcher. The MEPS is a commonly used instrument; within the LET literature, it is reported in 15% of studies. However, this tool was never designed for application in LET, and consequently, its domain structure may not reflect the experience of LET patients. The lack of data across all metric aspects highlights that this is likely to be an unsuitable instrument, yet its use appears to be increasing over time. The particular lack of data on the instrument’s interpretability in the context of LET exemplifies that though this is historically popular, researchers may struggle to justify its use. A similar scenario is present for the ASES-E score, which again lacks metric details in LET populations.

The remaining instruments scored below the required three out of five attribute scores for calculation of a composite score. They were all developed prior to 1986 and are clinician-rated. The lack of data on all of their metric properties implies that their use does not stand up to modern reporting requirements of outcome instruments [40, 41]. This is pertinent information due to the continued reporting of these instruments in contemporary literature.

This systematic review has focussed on the validity of condition-specific clinical rating scales, but it should be noted that the use of global impression scales or generic PROMs remains recommended as an adjunct. Although no history of explicit LET based validation has been undertaken, the use of such instruments as visual analogy pain and function scales or generic measures such as the 36-item Short Form survey (SF-36) or EuroQol 5-Dimension survey (EQ-5D) allows flexibility in results interpretation as they act as a common currency that allows aggregation and comparison across patient groups and health services, whilst the LET-specific rating scale imparts the detailed picture of a patient’s assessment of his/her own health [19].

### Limitations

This systematic review should be interpreted with reference to limitations inherent to its methodology. Firstly, our results are dependent on the information retrieved from the search strategy. It is important to note that the strategy was developed with reference to extensive protocols, and the largest health science databases (MEDLINE, Embase and CINAHL) were utilised and complemented with the addition of thesis searching and hand searching in recognised repositories. Furthermore, authors of the identified instruments were contacted and asked to confirm whether the list of manuscripts identified was as comprehensive as possible. Nevertheless, inherent in all search strategies is the possibility of missed or omitted evidence.

Secondly, the choice of the EMPRO tool itself should be scrutinised. Multiple attempts have been made to quantify the strength of evidence surrounding a set of instruments. The EMPRO tool was used owing to its emphasis on assessing the whole body of evidence relating to an instrument. We feel the validated output of a ‘score’ and recommendation is very beneficial to the clinician and researcher. The authors recognise that this may be complemented with the addition of the commonly cited COSMIN, which would scrutinise the methodological quality of the studies assessing the metric properties, rather than the instrument itself. This approach may be complementary, but to our knowledge, this method has not yet been reported.

Thirdly, it is recognised that our use of English language tools only limits the generalisability of our findings. However, we feel that the use of both non-English language instruments and data derived from cross-culturally adapted instruments imparts variables that the EMPRO tool was not conceived to deal with. Where the EMPRO provides comparative scores across instruments, the addition of information derived from a different cultural context is unhelpful for the researcher/clinician. Where adaptation of a tool is undertaken, certain aspects of its metric properties cannot be compared and should not be collated to complement the body of evidence [41, 42]. Strictly speaking, though all of the instruments were developed in the English language, the use of data from different English-speaking countries could be questioned. However, this group has identified that currently, pathology-specific and country-specific data for LET is not prevalent enough to allow such specific analysis. We would hope that the presented data encourage the exploration of culturally specific metric properties that would allow a detailed country-specific analysis in the future.

Fourthly, beyond the assessment of LET specific studies, this assessment derived some information from studies that contained a component of non-LET participants (Table 2). Therefore, contamination of our findings is possible as, in many instances, it is not possible to quantitatively extract the LET information and assess it in isolation from other pathologies. Here a pragmatic assessment was required to assess the psychometric strengths and weaknesses of the clinical rating system in the mixed cohort in which it is presented. Where possible, isolated subgroup analyses were taken as the predictive marker of psychometric performance, but this has to be tempered with the reduction of sample size in a mixed cohort. The authors feel that at present, though pathology-specific advice is highly sought after, it is a significant challenge in musculoskeletal health owing to the traditional use of region or joint-specific instruments. We advise our methods as a best possible route but would recommend that the reporting of pathology-specific details in all future development or assessment of musculoskeletal PROMs instruments will greatly enhance this process.

### Future

We hope that the presentation of information on both quality and distribution of use will compel researchers to carefully consider their instrument choice. Though this study reports the current strengths and weaknesses of LET instruments, it is important to comment on the changing landscape of outcome measure assessment in upper limb pathology. New novel instruments have been developed that integrate both patient-reported PROMs assessment and patient-reported objective function, including the German language Elbow Self-Assessment Score (ESAS) [43]. There is also an emergence of computer-based systems that use predictive algorithms to administer streamlined PROMs, easing data collection and analysis and decreasing participant burden. These systems offer great potential but are in the early stages of use in upper limb pathology [44]. Of note, the Patient-Reported Outcomes Measurement Information System (PROMIS), developed by the National Institutes of Health, is the largest computer-adaptive testing system but has no history of validation for the elbow region or specific elbow pathologies [44].

## Conclusion

This study is the first to provide a systematic evaluation of LET-specific PROMS instruments. The available evidence would currently support the use of the qDASH, DASH, PRTEE or OES instruments. Though the qDASH scored highest, we would advise that the choice of instrument should also depend upon the study’s particular requirements. We hope that the evidence presented for each metric attribute will facilitate the selection process. Future instrument development, particularly for those not meeting the recommended standards, can also be rationalised from the presented evidence. It is now clearly recognised that the choice of outcome instrument must be justified from both a validity and burden standpoint.

## Additional Files

Additional file 1: Search strategy. (DOCX 424 kb)

Additional file 2: List of included manuscripts identified and used in systematic assessment (DOCX 424 kb)

Additional file 3: EMPRO attribute and individual item scores for each outcome instrument. Item scores graded from 4 (strongly agree) to 1 (strongly disagree or no information). (DOCX 33 kb)

## Abbreviations

ASES-E: American Society of Shoulder and Elbow Surgeons-Elbow
COMET: Core Outcome Measures in Effectiveness Trials
COSMIN: Consensus-Based Standards for the Selection of health Measurement Instruments
CROM: Clinician-reported outcome measure
DASH: Disabilities of the Arm Shoulder and Hand
EMPRO: Evaluating measures of patient-reported outcomes
ESAS: Elbow Self-Assessment Score
HSS: Hospital for Special Surgery Score
ICHOM: Consortium for Health Outcomes Measurement
IQR: Interquartile range
LES: Liverpool Elbow Score
LET: Lateral elbow tendinopathy
MEPS: Mayo Elbow Performance Score
OES: Oxford Elbow Score
PRISMA: Preferred Reporting Items for Systematic Reviews and Meta-Analyses
PROM: Patient-reported outcome measure
PROQOLID: Patient-Reported Outcomes and Quality of Life Database
PRTEE: Patient-Rated Tennis Elbow Evaluation
qDASH: quick Disabilities of the Arm Shoulder and Hand
RCT: Randomised controlled trial
TEFS: Tennis Elbow Functional Scale
ULFI: Upper Limb Functional Index
