# Supplementary material for: Assessing Patient-Centred Outcomes in Lateral Elbow Tendinopathy: A Systematic Review and Standardised Comparison of English Language Clinical Rating Systems
Source: Sports Med Open. 2019 Mar 20;5:10. doi: 10.1186/s40798-019-0183-2 (PMC6426924; doi:10.1186/s40798-019-0183-2)
Supplement: Supplementary file 1 — Search strategy. (DOCX 424 kb) [file 40798_2019_183_MOESM1_ESM.docx]

Additional file 1

Search Strategy

Search Strategy – MEDLINE – Run 1/5/2017

Medline

- 1. exp Elbow/
  2. elbow.tw.
  3. exp Elbow joint/
  4. exp Tennis Elbow/
  5. epicondylitis.tw.
  6. common extensor origin.tw.
  7. epicondylalgia.tw.
  8. 1 or 2 or 3 or 4 or 5 or 6 or 7
  9. exp "Outcome Assessment (Health Care)"/
  10. (Outcome? adj2 assessment).tw.
  11. patient reported outcome?.tw.
  12. outcome? measure?.tw.
  13. exp health status/
  14. health status.tw.
  15. exp "quality of life"/
  16. quality of life.tw.
  17. (QL or QoL or HRQL or HRQoL).tw.
  18. (function* adj2 (status or psychological or mental or physical or social)).tw.
  19. disabilit*.tw.
  20. exp "Activities of Daily Living"/
  21. activities of daily living.tw.
  22. (wellbeing or well being).tw.
  23. exp happiness/
  24. (happi* or happy).tw.
  25. 9 or 10 or 11 or 12 or 13 or 14 or 15 or 16 or 17 or 18 or 19 or 20 or 21 or 22 or 23 or 24
  26. assessment.tw.
  27. index.tw.
  28. indices.tw.
  29. instrument?.tw.
  30. measure?.tw.
  31. profile?.tw.
  32. rating?.tw.
  33. report*.tw.
  34. scale?.tw.
  35. schedul*.tw.
  36. scor*.tw.
  37. exp health surveys/
  38. survey?.tw.
  39. 26 or 27 or 28 or 29 or 30 or 31 or 32 or 33 or 34 or 35 or 36 or 37 or 38
  40. (symptom? adj2 (assessment or index or indices or instrument? or measure? or profile? or rating? or report* or scale? or schedule? or scor* or survey?)).tw.
  41. 25 or 40
  42. exp Self-Assessment/
  43. self-assess*.tw.
  44. exp Questionnaires/
  45. questionnaire?.tw.
  46. self report*.tw.
  47. 42 or 43 or 44 or 45 or 46
  48. (Validation Studies or Comparative Study).pt. or exp psychometrics/ or psychometr*.tw. or clinimetr*.tw. or clinometr*.tw. or exp observer variation/ or observer variation.tw. or exp Health Status Indicators/ or exp reproducibility of results/ or reproducib*.tw. or exp discriminant analysis/ or reliab*.tw. or unreliab*.tw. or valid*.tw. or coefficient.tw. or homogeneity.tw. or homogeneous.tw. or internal consistency.tw. or (cronbach* and (alpha or alphas)).tw. or (item and (correlation* or selection* or reduction*)).tw. or agreement.tw. or precision.tw. or imprecision.tw. or precise values.tw. or test-retest.tw. or (test and retest).tw. or (reliab* and (test or retest)).tw. or stability.tw. or interrater.tw. or inter-rater.tw. or intrarater.tw. or intra-rater.tw. or intertester.tw. or inter-tester.tw. or intratester.tw. or intra-tester.tw. or interobserver.tw. or inter-observer.tw. or intraobserver.tw. or intraobserver.tw. or intertechnician.tw. or inter-technician.tw. or intratechnician.tw. or intra-technician.tw. or interexaminer.tw. or inter-examiner.tw. or intraexaminer.tw. or intra-examiner.tw. or interassay.tw. or inter-assay.tw. or intraassay.tw. or intra-assay.tw. or interindividual.tw. or inter-individual.tw. or intraindividual.tw. or intra-individual.tw. or interparticipant.tw. or inter-participant.tw. or intraparticipant.tw. or intra-participant.tw. or kappa.tw. or kappa*.tw. or kappas.tw. or repeatab*.tw. or ((replicab* or repeated) and (measure or measures or findings or result or results or test or tests)).tw. or concordance.tw. or (intraclass and correlation*).tw. or discriminative.tw. or known group.tw. or factor analysis.tw. or factor analyses.tw. or dimension*.tw. or subscale*.tw. or (multitrait and scaling and (analysis or analyses)).tw. or item discriminant.tw. or interscale correlation*.tw. or error.tw. or errors.tw. or individual variability.tw. or (variability and (analysis or values)).tw. or (uncertainty and (measurement or measuring)).tw. or standard error of measurement.tw. or sensitiv*.tw. or responsive*.tw. or ((minimal or minimally or clinical or clinically) and (important or significant or detectable) and (change or difference)).tw. or (small* and (real or detectable) and (change or difference)).tw. or meaningful change.tw. or ceiling effect.tw. or floor effect.tw. or Item response model.tw. or IRT.tw. or Rasch.tw. or Differential item functioning.tw. or DIF.tw. or computer adaptive testing.tw. or item bank.tw. or cross-cultural equivalence.tw.
  49. 39 or 47 or 48
  50. 41 and 49
  51. (Oxford elbow score or Liverpool Elbow Score or Elbow Self-Assessment Score or Elbow Function Assessment or (American Shoulder and Elbow Surgeons-elbow) or (Modified American Shoulder and Elbow Surgeons) or Mayo Elbow Performance Score or Hospital for Special Surgery score or Hospital for Special Surgery short version or patient-rated elbow evaluation or Patient-Rated Tennis Elbow Evaluation or Elbow Functional Assessment or (Disabilities of the Arm, Shoulder and Hand questionnaire) or subjective elbow value or (Broberg and Morrey) or Ewald).mp. or Pritchard.tw. [mp=ti, ab, ot, nm, hw, kf, px, rx, ui, tn, dm, mf, dv, kw]
  52. (OES or LES or ESAS or ASES or ASES-e or MEP or PREE or PRTEE or EFA or DASH or quickDASH).mp. [mp=ti, ab, ot, nm, hw, kf, px, rx, ui, tn, dm, mf, dv, kw]
  53. 8 and 52
  54. 8 and 50
  55. 51 or 53 or 54
  56. exp ANIMALS/ not humans.sh.
  57. 55 not 56
