# Supplementary material for: Assessing Patient-Centred Outcomes in Lateral Elbow Tendinopathy: A Systematic Review and Standardised Comparison of English Language Clinical Rating Systems
Source: Sports Med Open. 2019 Mar 20;5:10. doi: 10.1186/s40798-019-0183-2 (PMC6426924; doi:10.1186/s40798-019-0183-2)
Supplement: Supplementary file 2 — List of included manuscripts identified and used in systematic assessment (DOCX 424 kb) [file 40798_2019_183_MOESM2_ESM.docx]

Additional file 2

List of Included manuscripts identified and used in systematic assessment

Key: A&C = Andrews and Carson, ASES-e = American Shoulder and Elbow Score-e, DASH = Disabilities of the Arm, Shoulder and Hand, HSS = Hospital for Special Surgery, LES = Liverpool Elbow Score, MEPS = Mayo Elbow Performance Score, OES = Oxford Elbow Score, PRTEE = Patient-Rated Tennis Elbow Evaluation, qDASH = quick Disabilities of the Arm Shoulder and Hand, R&M = Roles and Maudsley, TEFS = Tennis Elbow Functional Score, ULFI = Upper Limb Functional Index

| Instrument | Manuscript identified as reporting development or metric properties | Manuscripts identified as reporting cross-cultural adaptation in LET population | Manuscript identified as reporting instrument use (Internationally) | Manuscript identified as reporting instrument use (English speaking population) |
| --- | --- | --- | --- | --- |
| A & C | (1) |  | (2) (3) | (2) (3) |
| ASES-e | (4) (5) (6) |  | (7) (8) (9) (10) (11) | (12) (10) (11) (7) (8) |
| DASH | (13) (14) (15) (16) (17) (18) (19) (20) (21) (6) (22) (23) (24) (25) (26) (27) | (28) (29) (30) (31) (32) (33) (34) | (35) (36) (37) (38) (39) (40) (41) (42) (43) (44) (45) (46) (47) (48) (49) (50) (51) (52) (53) (54) (55) (56) (57) (58) (59) (60) (61) (62) (63) (64) (65) (66) (67) (68) (69) (70) (71) (72) (73) (74) (75) (76) (77) (78) (79) (80) (81) (82) (83) (84) (85) (86) (87) (88) (89) (70) (90) (91) (92) (93) (94) | (35) (36) (37) (39) (41) (42) (43) (45) (46) (52) (54) (55) (59) (60) (61) (66) (68) (73) (74) (78) (79) (89) (38) |
| HSS | (95) (96) (27) |  | (97) | (97) |
| LES | (24) |  | (98) |  |
| MEPS | (99) (4) (13) (15) (16) (27) | (100) | (101) (102) (103) (7) (104) (105) (106) (107) (108) (90) (93) (109) (110) (97) (113) (114) (115) (116) (117) (2) (71) (94) | (101) (7) (104) (106) (97) (2) (66) (115) (10) |
| MORREY | (118) (27) |  | (119) (120) (121) (94) |  |
| Nirschl | (122) (9) |  | (123) (124) (8) (125) (126) (127) (128) (129) (130) (9) (131) (110) (132) (133) (134) (92) | (127) (128) (130) (135) (8) (136) (12) |
| OES | (13) (14) (15) (16) (137) | (138) (139) (140) (141) (142) (143) | (144) (145) (71) (146)  (78) | (61) (78) |
| PRTEE | (147) (22) (148) (149) (23) (150) (151) | (152) (153) (154) (155) (32) (33) (34) (156) (157) | (10) (89) (158) (159) (160) (161) (162) (163) (164) (165) (166) (167) (168) (169) (170) (171) (172) (173) (174) (175) (176) (177) (178) (179) (180) (181) (182) (183) (184) (185) (186) (183) (187) (188) (189) (190) (191) (160) (192) (10) (49) (59) (93) (193) (194) (195) (196) (197) (198) (82) (133) (199) | (89) (192) (158) (159) (160) (200) (160) (201) (202) (169) (188) (173) (175) (177) (190) (181) (187) (194) (10) (59) (43) |
| QDASH | (203) (204) (21) (205) (206) (26) (207) | (152) (139) (30) | (131) (113) (208) (209) (210) (211) (115) (212) (213) (214) (215) (216) (217) (218) (219) (220) (221) (134) | (216) (211) (115) (214) (219) |
| R & M | (222) (23) | (155) (32) | (223) (224) (225) (226) (227) (121) (228) (226) (229) (230) (231) (232) (132) (213) (233) (199) | (227) (232) |
| TEFS | (234) |  | (235) (236) |  |
| ULFI | (237) |  | (238) (117) (239) (240) (214) | (239) (216) (214) |
| Verhaar | (241) |  | (242) (243) (244) (245) (246) (9) | (12) |

1. Andrews JR, St Pierre RK, Carson Jr WG. Arthroscopy of the elbow. Clinics in sports medicine. 1986;5(4):653-62.

2. Dzugan SS, Savoie FH, 3rd, Field LD, O'Brien MJ, You Z. Acute radial ulno-humeral ligament injury in patients with chronic lateral epicondylitis: an observational report. J Shoulder Elbow Surg. 2012;21(12):1651-5.

3. Szabo SJ, Savoie FH, Field LD, Ramsey JR, Hosemann CD. Tendinosis of the extensor carpi radialis brevis: an evaluation of three methods of operative treatment. Journal of shoulder and elbow surgery. 2006;15(6):721-7.

4. Cusick MC, Bonnaig NS, Azar FM, Mauck BM, Smith RA, Throckmorton TW. Accuracy and reliability of the Mayo Elbow Performance Score. J Hand Surg Am. 2014;39(6):1146-50.

5. King GJ, Richards RR, Zuckerman JD, Blasier R, Dillman C, Friedman RJ, et al. A standardized method for assessment of elbow function. Research Committee, American Shoulder and Elbow Surgeons. Journal of shoulder and elbow surgery / American Shoulder and Elbow Surgeons [et al]. 1999;8(4):351-4.

6. MacDermid JC. Outcome evaluation in patients with elbow pathology: issues in instrument development and evaluation. Journal of Hand Therapy. 2001;14(2):105-14.

7. Garg R, Adamson GJ, Dawson PA, Shankwiler JA, Pink MM. A prospective randomized study comparing a forearm strap brace versus a wrist splint for the treatment of lateral epicondylitis. J Shoulder Elbow Surg. 2010;19(4):508-12.

8. Schipper ON, Dunn JH, Ochiai DH, Donovan JS, Nirschl RP. Nirschl surgical technique for concomitant lateral and medial elbow tendinosis: a retrospective review of 53 elbows with a mean follow-up of 11.7 years. Am J Sports Med. 2011;39(5):972-6.

9. Dunn JH, Kim JJ, Davis L, Nirschl RP. Ten-to 14-year follow-up of the Nirschl surgical technique for lateral epicondylitis. The American journal of sports medicine. 2008;36(2):261-6.

10. Grewal R, MacDermid JC, Shah P, King GJ. Functional outcome of arthroscopic extensor carpi radialis brevis tendon release in chronic lateral epicondylitis. J Hand Surg Am. 2009;34(5):849-57.

11. Hechtman KS, Uribe JW, Botto-vanDemden A, Kiebzak GM. Platelet-rich plasma injection reduces pain in patients with recalcitrant epicondylitis. Orthopedics. 2011;34(2):92.

12. Dunn JH, Kim JJ, Davis L, Nirschl RP. Ten- to 14-year follow-up of the Nirschl surgical technique for lateral epicondylitis. Am J Sports Med. 2008;36(2):261-6.

13. Dawson J, Doll H, Boller I, Fitzpatrick R, Little C, Rees J, et al. The development and validation of a patient-reported questionnaire to assess outcomes of elbow surgery. J Bone Joint Surg Br. 2008;90(4):466-73.

14. Dawson J, Doll H, Boller I, Fitzpatrick R, Little C, Rees J, et al. Comparative responsiveness and minimal change for the Oxford Elbow Score following surgery. Qual Life Res. 2008;17(10):1257-67.

15. Dawson J, Doll H, Boller I, Fitzpatrick R, Little C, Rees J, et al. Factors associated with satisfaction in patients undergoing elbow surgery: a prospective study. J Shoulder Elbow Surg. 2010;19(5):635-44.

16. Dawson J, Doll H, Boller I, Fitzpatrick R, Little C, Rees J, et al. Specificity and responsiveness of patient-reported and clinician-rated outcome measures in the context of elbow surgery, comparing patients with and without rheumatoid arthritis. Orthop Traumatol-Sur. 2012;98(6):652-8.

17. Gummesson C, Atroshi I, Ekdahl C. The disabilities of the arm, shoulder and hand (DASH) outcome questionnaire: longitudinal construct validity and measuring self-rated health change after surgery. BMC Musculoskelet Disord. 2003;4(1):11.

18. Hudak PL, Amadio PC, Bombardier C, Beaton D, Cole D, Davis A, et al. Development of an upper extremity outcome measure: the DASH (disabilities of the arm, shoulder, and hand). American journal of industrial medicine. 1996;29(6):602-8.

19. Hunsaker FG, Cioffi DA, Amadio PC, Wright JG, Caughlin B. The American academy of orthopaedic surgeons outcomes instruments. J Bone Joint Surg Am. 2002;84(2):208-15.

20. Janssen S, De Smet L. Responsiveness of the DASH questionnaire for surgically treated tennis elbow. Acta Chir Belg. 2008;108(5):583-5.

21. Kachooei AR, Moradi A, Janssen SJ, Ring D. The influence of dominant limb involvement on DASH and QuickDASH. Hand (N Y). 2015;10(3):512-5.

22. Newcomer KL, Martinez-Silvestrini JA, Schaefer MP, Gay RE, Arendt KW. Sensitivity of the Patient-rated Forearm Evaluation Questionnaire in lateral epicondylitis. Journal of Hand Therapy. 2005;18(4):400-73 8p.

23. Rompe JD, Overend TJ, MacDermid JC. Validation of the Patient-rated Tennis Elbow Evaluation Questionnaire. J Hand Ther. 2007;20(1):3-10; quiz 1.

24. Sathyamoorthy P, Kemp G, Rawal A, Rayner V, Frostick S. Development and validation of an elbow score. Rheumatology. 2004;43(11):1434-40.

25. Schmitt JS, Di Fabio RP. Reliable change and minimum important difference (MID) proportions facilitated group responsiveness comparisons using individual threshold criteria. J Clin Epidemiol. 2004;57(10):1008-18.

26. Sorensen AA, Howard D, Tan WH, Ketchersid J, Calfee RP. Minimal clinically important differences of 3 patient-rated outcomes instruments. The Journal of hand surgery. 2013;38(4):641-9.

27. Turchin DC, Beaton DE, Richards RR. Validity of observer-based aggregate scoring systems as descriptors of elbow pain, function, and disability. The Journal of bone and joint surgery American volume. 1998;80(2):154-62.

28. Atroshi I, Gummesson C, Andersson B, Dahlgren E, Johansson A. The disabilities of the arm, shoulder and hand (DASH) outcome questionnaire: reliability and validity of the Swedish version evaluated in 176 patients. Acta Orthopaedica Scandinavica. 2000;71(6):613-8.

29. Franchignoni F, Giordano A, Sartorio F, Vercelli S, Pascariello B, Ferriero G. Suggestions for refinement of the Disabilities of the Arm, Shoulder and Hand Outcome Measure (DASH): a factor analysis and Rasch validation study. Arch Phys Med Rehabil. 2010;91(9):1370-7.

30. Franchignoni F, Vercelli S, Giordano A, Sartorio F, Bravini E, Ferriero G. Minimal clinically important difference of the disabilities of the arm, shoulder and hand outcome measure (DASH) and its shortened version (QuickDASH). journal of orthopaedic & sports physical therapy. 2014;44(1):30-9.

31. Mousavi SJ, Parnianpour M, Abedi M, Askary-Ashtiani A, Karimi A, Khorsandi A, et al. Cultural adaptation and validation of the Persian version of the Disabilities of the Arm, Shoulder and Hand (DASH) outcome measure. Clin Rehabil. 2008;22(8):749-57.

32. Nilsson P, Baigi A, Marklund B, Mansson J. Cross-cultural adaptation and determination of the reliability and validity of PRTEE-S (Patientskattad Utvardering av Tennisarmbage), a questionnaire for patients with lateral epicondylalgia, in a Swedish population. BMC Musculoskelet Disord. 2008;9:79.

33. Stasinopoulos D, Papadopoulos C, Antoniadou M, Nardi L. Greek adaptation and validation of the Patient-Rated Tennis Elbow Evaluation (PRTEE). J Hand Ther. 2015;28(3):286-90; quiz 91.

34. van Ark M, Zwerver J, Diercks RL, van den Akker-Scheek I. Cross-cultural adaptation and reliability and validity of the Dutch Patient-Rated Tennis Elbow Evaluation (PRTEE-D). BMC Musculoskelet Disord. 2014;15:270.

35. Davidson JH, Vandervoort A, Lessard L, Miller L. The effect of acupuncture versus ultrasound on pain level, grip strength and disability in individuals with lateral epicondylitis: a pilot study. Physiotherapy Canada. 2001;53(3):195-211 9p.

36. Melikyan EY, Shahin E, Miles J, Bainbridge LC. Extracorporeal shock-wave treatment for tennis elbow: a randomised double-blind study. Journal of Bone & Joint Surgery, British Volume. 2003;85B(6):852-5 4p.

37. Dunkow PD, Jatti M, Muddu BN. A comparison of open and percutaneous techniques in the surgical treatment of tennis elbow. Journal of Bone & Joint Surgery, British Volume. 2004;86B(5):701-4 4p.

38. Waugh EJ, Jaglal SB, Davis AM. Computer use associated with poor long-term prognosis of conservatively managed lateral epicondylalgia. The Journal of orthopaedic and sports physical therapy. 2004;34(12):770-80.

39. Waugh EJ, Jaglal SB, Davis AM, Tomlinson G, Verrier MC. Factors associated with prognosis of lateral epicondylitis after 8 weeks of physical therapy. Arch Phys Med Rehab. 2004;85(2):308-18.

40. Spacca G, Necozione S, Cacchio A. Radial shock wave therapy for lateral epicondylitis: a prospective randomised controlled single-blind study. Europa medicophysica. 2005;41(1):17-25.

41. Tasto JP, Cummings J, Medlock V, Hardesty R, Amiel D. Microtenotomy using a radiofrequency probe to treat lateral epicondylitis. Arthroscopy : the journal of arthroscopic & related surgery : official publication of the Arthroscopy Association of North America and the International Arthroscopy Association. 2005;21(7):851-60.

42. Thornton SJ, Rogers JR, Prickett WD, Dunn WR, Allen AA, Hannafin JA. Treatment of recalcitrant lateral epicondylitis with suture anchor repair. The American journal of sports medicine. 2005;33(10):1558-64.

43. Alizadehkhaiyat O, Fisher AC, Kemp GJ, Frostick SP. Pain, functional disability, and psychologic status in tennis elbow. Clin J Pain. 2007;0(6):482-9.

44. Lam LK, Cheing GL. Effects of 904-nm low-level laser therapy in the management of lateral epicondylitis: a randomized controlled trial. Photomed Laser Surg. 2007;25(2):65-71.

45. Brockenbrough G. No significant differences seen with steroids vs. placebo for lateral elbow pain. Orthopedics Today. 2008;28(5):63-4 2p.

46. Lindenhovius A, Henket M, Gilligan BP, Lozano-Calderon S, Jupiter JB, Ring D. Injection of dexamethasone versus placebo for lateral elbow pain: a prospective, double-blind, randomized clinical trial. J Hand Surg Am. 2008;33(6):909-19.

47. Oskarsson E, Piehl Aulin K, Gustafsson BE, Pettersson K. Improved intramuscular blood flow and normalized metabolism in lateral epicondylitis after botulinum toxin treatment. Scand J Med Sci Sports. 2009;19(3):323-8.

48. Akin C, Oken O, Koseoglu BF. Short-Term Effectiveness of Ultrasound Treatment in Patients with Lateral Epicondylitis: Randomized, Single-Blind, Placebo-Controlled, Prospective Study. Turk J Rheumatol. 2010;25(2):50-5.

49. Emanet SK, Altan LI, Yurtkuran M. Investigation of the effect of GaAs laser therapy on lateral epicondylitis. Photomed Laser Surg. 2010;28(3):397-403.

50. Gong HS, Chung MS, Kang ES, Oh JH, Lee YH, Baek GH. Musculofascial lengthening for the treatment of patients with medial epicondylitis and coexistent ulnar neuropathy. J Bone Joint Surg Br. 2010;92(6):823-7.

51. Peerbooms JC, Sluimer J, Bruijn DJ, Gosens T. Positive effect of an autologous platelet concentrate in lateral epicondylitis in a double-blind randomized controlled trial: platelet-rich plasma versus corticosteroid injection with a 1-year follow-up. Am J Sports Med. 2010;38(2):255-62.

52. Tyler TF, Thomas GC, Nicholas SJ, McHugh MP. Addition of isolated wrist extensor eccentric exercise to standard treatment for chronic lateral epicondylosis: a prospective randomized trial. J Shoulder Elbow Surg. 2010;19(6):917-22.

53. Backer M, Ludtke R, Afra D, Cesur O, Langhorst J, Fink M, et al. Effectiveness of leech therapy in chronic lateral epicondylitis: a randomized controlled trial. Clin J Pain. 2011;27(5):442-7.

54. Bellapianta J, Swartz F, Lisella J, Czajka J, Neff R, Uhl R. Randomized prospective evaluation of injection techniques for the treatment of lateral epicondylitis. Orthopedics. 2011;34(11):e708-12.

55. Carayannopoulos A, Borg-Stein J, Sokolof J, Meleger A, Rosenberg D. Prolotherapy versus corticosteroid injections for the treatment of lateral epicondylosis: a randomized controlled trial. PM R. 2011;3(8):706-15.

56. Gosens T, Peerbooms JC, van Laar W, den Oudsten BL. Ongoing positive effect of platelet-rich plasma versus corticosteroid injection in lateral epicondylitis: a double-blind randomized controlled trial with 2-year follow-up. Am J Sports Med. 2011;39(6):1200-8.

57. Othman AM. Arthroscopic versus percutaneous release of common extensor origin for treatment of chronic tennis elbow. Arch Orthop Trauma Surg. 2011;131(3):383-8.

58. Peterson M, Butler S, Eriksson M, Svardsudd K. A randomized controlled trial of exercise versus wait-list in chronic tennis elbow (lateral epicondylosis). Ups J Med Sci. 2011;116(4):269-79.

59. Wolf JM, Ozer K, Scott F, Gordon MJ, Williams AE. Comparison of autologous blood, corticosteroid, and saline injection in the treatment of lateral epicondylitis: a prospective, randomized, controlled multicenter study. J Hand Surg Am. 2011;36(8):1269-72.

60. Agostinucci J, McLinden J, Cherry E. The effect of cryotherapy and exercise on lateral epicondylitis: a controlled randomised study. International Journal of Therapy & Rehabilitation. 2012;19(11):641-50 10p.

61. Nazar M, Lipscombe S, Morapudi S, Tuvo G, Kebrle R, Marlow W, et al. Percutaneous tennis elbow release under local anaesthesia. Open Orthop J. 2012;6:129-32.

62. Okcu G, Erkan S, Senturk M, Ozalp RT, Yercan HS. Evaluation of injection techniques in the treatment of lateral epicondylitis: a prospective randomized clinical trial. Acta Orthop Traumatol Turc. 2012;46(1):26-9.

63. Küçükşen S, Yilmaz H, Sallı A, Uğurlu H. Muscle Energy Technique Versus Corticosteroid Injection for Management of Chronic Lateral Epicondylitis: Randomized Controlled Trial With 1-Year Follow-up. 2013;94(11):2068-74.

64. Martin JI, Merino J, Atilano L, Areizaga LM, Gomez-Fernandez MC, Burgos-Alonso N, et al. Platelet-rich plasma (PRP) in chronic epicondylitis: study protocol for a randomized controlled trial. Trials. 2013;14:410.

65. Rhyou IH, Kim KW. Is posterior synovial plica excision necessary for refractory lateral epicondylitis of the elbow? Clinical orthopaedics and related research. 2013;471(1):284-90.

66. Shahid M, Wu F, Deshmukh SC. Operative treatment improves patient function in recalcitrant medial epicondylitis. Ann R Coll Surg Engl. 2013;95(7):486-8.

67. Kwon BC, Kwon YS, Bae KJ. The Fascial Elevation and Tendon Origin Resection Technique for the Treatment of Chronic Recalcitrant Medial Epicondylitis. Am J Sports Med. 2014;42(7):1731-7.

68. Tyler TF, Nicholas SJ, Schmitt BM, Mullaney M, Hogan DE. Clinical outcomes of the addition of eccentrics for rehabilitation of previously failed treatments of golfers elbow. Int J Sports Phys Ther. 2014;9(3):365-70.

69. Valera-Garrido F, Minaya-Munoz F, Medina-Mirapeix F. Ultrasound-guided percutaneous needle electrolysis in chronic lateral epicondylitis: short-term and long-term results. Acupunct Med. 2014;32(6):446-54.

70. Wada T, Moriya T, Iba K, Ozasa Y, Sonoda T, Aoki M, et al. Functional outcomes after arthroscopic treatment of lateral epicondylitis. J Orthop Sci. 2009;14(2):167-74.

71. Gautam VK, Verma S, Batra S, Bhatnagar N, Arora S, Verma S, et al. Platelet-rich plasma versus corticosteroid injection for recalcitrant lateral epicondylitis: clinical and ultrasonographic evaluation. Journal of Orthopaedic Surgery (10225536). 2015;23(1):1-5 p.

72. Lebiedzinski R, Synder M, Buchcic P, Polguj M, Grzegorzewski A, Sibinski M. A randomized study of autologous conditioned plasma and steroid injections in the treatment of lateral epicondylitis. International Orthopaedics. 2015;39(11):2199-203.

73. Ruch DS, Orr SB, Richard MJ, Leversedge FJ, Mithani SK, Laino DK. A comparison of debridement with and without anconeus muscle flap for treatment of refractory lateral epicondylitis. Journal of Shoulder and Elbow Surgery. 2015;24(2):236-41.

74. Sevier TL, Stegink-Jansen CW. Astym treatment vs. eccentric exercise for lateral elbow tendinopathy: a randomized controlled clinical trial. PeerJ. 2015;3:e967.

75. Tetschke E, Rudolf M, Lohmann CH, Starke C. Autologous proliferative therapies in recalcitrant lateral epicondylitis. Am J Phys Med Rehabil. 2015;94(9):696-706.

76. Yadav R, Kothari SY, Borah D. Comparison of Local Injection of Platelet Rich Plasma and Corticosteroids in the Treatment of Lateral Epicondylitis of Humerus. J Clin Diagn Res. 2015;9(7):RC05-7.

77. Akkurt E, Kucuksen S, Yilmaz H, Parlak S, Salli A, Karaca G. Long term effects of high intensity laser therapy in lateral epicondylitis patients. Lasers Med Sci. 2016;31(2):249-53.

78. Grawe BM, Fabricant PD, Chin CS, Allen AA, DePalma BJ, Dines DM, et al. Clinical Outcomes After Suture Anchor Repair of Recalcitrant Medial Epicondylitis. Orthopedics. 2016;39(1):e104-7.

79. Seng C, Mohan PC, Koh SB, Howe TS, Lim YG, Lee BP, et al. Ultrasonic Percutaneous Tenotomy for Recalcitrant Lateral Elbow Tendinopathy: Sustainability and Sonographic Progression at 3 Years. Am J Sports Med. 2016;44(2):504-10.

80. Liu YS, Gadau M, Zhang GX, Liu H, Wang FC, Zaslawski C, et al. Acupuncture Treatment of Lateral Elbow Pain: A Nonrandomized Pilot Study. Evid Based Complement Alternat Med. 2016;2016:8182071.

81. Najafi M, Arazpour M, Aminian G, Curran S, Madani SP, Hutchins SW. Effect of a new hand-forearm splint on grip strength, pain, and function in patients with tennis elbow. Prosthet Orthot Int. 2016;40(3):363-8.

82. Palacio EP, Schiavetti RR, Kanematsu M, Ikeda TM, Mizobuchi RR, Galbiatti JA. Effects of platelet-rich plasma on lateral epicondylitis of the elbow: prospective randomized controlled trial. Revista Brasileira de Ortopedia. 2016;51(1):90-5.

83. Hsu CY, Lee KH, Huang HC, Chang ZY, Chen HY, Yang TH. Manipulation Therapy Relieved Pain More Rapidly Than Acupuncture among Lateral Epicondylalgia (Tennis Elbow) Patients: A Randomized Controlled Trial with 8-Week Follow-Up. Evid Based Complement Alternat Med. 2016;2016:3079247.

84. Nascimento AT, Claudio GK. Arthroscopic surgical treatment of recalcitrant lateral epicondylitis - A series of 47 cases. Revista Brasileira de Ortopedia. 2017;52(1):46-51.

85. Schoffl V, Willauschus W, Sauer F, Kupper T, Schoffl I, Lutter C, et al. Autologous Conditioned Plasma Versus Placebo Injection Therapy in Lateral Epicondylitis of the Elbow: A Double Blind, Randomized Study. Sportverletz Sportschaden. 2017;31(1):31-6.

86. Yang TH, Huang YC, Lau YC, Wang LY. Efficacy of Radial Extracorporeal Shock Wave Therapy on Lateral Epicondylosis, and Changes in the Common Extensor Tendon Stiffness with Pretherapy and Posttherapy in Real-Time Sonoelastography: A Randomized Controlled Study. Am J Phys Med Rehabil. 2017;96(2):93-100.

87. Martinez-Cervera FV, Olteanu TE, Gil-Martinez A, Diaz-Pulido B, Ferrer-Pena R. Influence of expectations plus mobilization with movement in patient with lateral epicondylalgia: a pilot randomized controlled trial. Journal of Exercise Rehabilitation. 2017;13(1):101-9.

88. Shakeri H, Soleimanifar M, Arab AM, Hamneshin Behbahani S. The effects of KinesioTape on the treatment of lateral epicondylitis. J Hand Ther. 2017;27:27.

89. Martinez-Silvestrini JA, Newcomer KL, Gay RE, Schaefer MP, Kortebein P, Arendt KW. Chronic lateral epicondylitis: comparative effectiveness of a home exercise program including stretching alone versus stretching supplemented with eccentric or concentric strengthening. Journal of Hand Therapy. 2005;18(4):411-73 11p.

90. Sauvage A, Nedellec G, Brulard C, Gaumet G, Mesnil P, Fontaine C, et al. [Arthroscopic treatment of lateral epicondylitis: a prospective study on 14 cases]. Chir Main. 2013;32(2):80-4.

91. Oki G, Iba K, Sasaki K, Yamashita T, Wada T. Time to functional recovery after arthroscopic surgery for tennis elbow. J Shoulder Elbow Surg. 2014;23(10):1527-31.

92. Han SH, Lee JK, Kim HJ, Lee SH, Kim JW, Kim TS. The result of surgical treatment of medial epicondylitis: analysis with more than a 5-year follow-up. J Shoulder Elbow Surg. 2016;25(10):1704-9.

93. Babaqi AA, Kotb MM, Said HG, AbdelHamid MM, ElKady HA, ElAssal MA. Short-term evaluation of arthroscopic management of tennis elbow; including resection of radio-capitellar capsular complex. J Orthop. 2014;11(2):82-6.

94. Jimenez I, Marcos-Garcia A, Muratore-Moreno G, Medina J. [Four surgical tips in the treatment of epicondylitis]. Rev. 2016;60(1):38-43.

95. Figgie MP, Inglis AE, Mow CS, Figgie HE, 3rd. Total elbow arthroplasty for complete ankylosis of the elbow. J Bone Joint Surg Am. 1989;71(4):513-20.

96. Inglis AE, Pellicci PM. Total elbow replacement. The Journal of Bone & Joint Surgery. 1980;62(8):1252-8.

97. Coleman B, Quinlan JF, Matheson JA. Surgical treatment for lateral epicondylitis: a long-term follow-up of results. J Shoulder Elbow Surg. 2010;19(3):363-7.

98. Thanasas C, Papadimitriou G, Charalambidis C, Paraskevopoulos I, Papanikolaou A. Platelet-rich plasma versus autologous whole blood for the treatment of chronic lateral elbow epicondylitis: a randomized controlled clinical trial. Am J Sports Med. 2011;39(10):2130-4.

99. Morrey B, Adams R. Semiconstrained arthroplasty for the treatment of rheumatoid arthritis of the elbow. J Bone Joint Surg Am. 1992;74(4):479-90.

100. Celik D. Psychometric properties of the Mayo Elbow Performance Score. Rheumatol Int. 2015;35(6):1015-20.

101. Baker CL, Jr., Baker CL, 3rd. Long-term follow-up of arthroscopic treatment of lateral epicondylitis. Am J Sports Med. 2008;36(2):254-60.

102. Moon YL, Jo SH, Song CH, Park G, Lee HJ, Jang SJ. Autologous bone marrow plasma injection after arthroscopic debridement for elbow tendinosis. Ann Acad Med Singapore. 2008;37(7):559-63.

103. Yan H, Cui GQ, Liu YL, Xiao J, Yang YP, Ao YF. [A randomized comparison of open and arthroscopic Nirschl debridement for refractory lateral epicondylitis]. Zhonghua Wai Ke Za Zhi. 2009;47(12):888-91.

104. Lattermann C, Romeo AA, Anbari A, Meininger AK, McCarty LP, Cole BJ, et al. Arthroscopic debridement of the extensor carpi radialis brevis for recalcitrant lateral epicondylitis. J Shoulder Elbow Surg. 2010;19(5):651-6.

105. Kim JW, Chun CH, Shim DM, Kim TK, Kweon SH, Kang HJ, et al. Arthroscopic treatment of lateral epicondylitis: comparison of the outcome of ECRB release with and without decortication. Knee Surg Sports Traumatol Arthrosc. 2011;19(7):1178-83.

106. Seitz WH, Jr., Lall A. Treatment of chronic tennis elbow with radiofrequency coblation and localized arthroscopic synovectomy. Current Orthopaedic Practice. 2012;23(6):621-4 4p.

107. Deng Y, Tang K, Li H, Zhang J, Xie M, Cao H, et al. [Short-term effectiveness of suture anchor after debridement of extensor tendon insertion for recalcitrant lateral epicondylitis]. Zhongguo Xiu Fu Chong Jian Wai Ke Za Zhi. 2013;27(1):1-6.

108. Meknas K, Al Hassoni TN, Odden-Miland A, Castillejo M, Kartus J. Medium-Term Results After Treatment of Recalcitrant Lateral Epicondylitis: A Prospective, Randomized Study Comparing Open Release and Radiofrequency Microtenotomy. Orthop J Sports Med. 2013;1(4):2325967113505433.

109. Notarnicola A, Quagliarella L, Sasanelli N, Maccagnano G, Fracella MR, Forcignano MI, et al. Effects of extracorporeal shock wave therapy on functional and strength recovery of handgrip in patients affected by epicondylitis. Ultrasound Med Biol. 2014;40(12):2830-40.

110. Behera P, Dhillon M, Aggarwal S, Marwaha N, Prakash M. Leukocyte-poor platelet-rich plasma versus bupivacaine for recalcitrant lateral epicondylar tendinopathy. J Orthop Surg (Hong Kong). 2015;23(1):6-10.

111. Luo KM, Hou Z, Bu AX, Yang L. [Electroacupuncture in combination with rehabilitation for treatment of motor impairment after elbow operation]. Zhongguo Zhen Jiu. 2010;30(7):559-62.

112. Vinod AV, Ross G. An effective approach to diagnosis and surgical repair of refractory medial epicondylitis. J Shoulder Elbow Surg. 2015;24(8):1172-7.

113. Lin CL, Lee JS, Su WR, Kuo LC, Tai TW, Jou IM. Clinical and ultrasonographic results of ultrasonographically guided percutaneous radiofrequency lesioning in the treatment of recalcitrant lateral epicondylitis. Am J Sports Med. 2011;39(11):2429-35.

114. Shahid M, Wu F, Deshmukh SC. Operative treatment improves patient function in recalcitrant medial epicondylitis. The Annals of The Royal College of Surgeons of England. 2013;95(7):486-8.

115. Barnes DE, Beckley JM, Smith J. Percutaneous ultrasonic tenotomy for chronic elbow tendinosis: a prospective study. J Shoulder Elbow Surg. 2015;24(1):67-73.

116. Ertem K, Ergen E, Yologlu S. Functional outcomes of arthroscopic treatment of lateral epicondylitis. Acta Orthop Traumatol Turc. 2015;49(5):471-7.

117. Yoon JP, Chung SW, Yi JH, Lee BJ, Jeon IH, Jeong WJ, et al. Prognostic Factors of Arthroscopic Extensor Carpi Radialis Brevis Release for Lateral Epicondylitis. Arthroscopy. 2015;31(7):1232-7.

118. Broberg MA, Morrey B. Results of delayed excision of the radial head after fracture. J Bone Joint Surg Am. 1986;68(5):669-74.

119. Seegenschmiedt MH, Keilholz L, Martus P, Kuhr M, Wichmann G, Sauer R. [Epicondylopathia humeri. The indication for, technic and clinical results of radiotherapy]. Epicondylopathia humeri Indikation, Technik, klinische Ergebnisse der Radiotherapie. 1997;173(4):208-18.

120. Seegenschmiedt MH, Keilholz L. Epicondylopathia humeri (EPH) and peritendinitis humeroscapularis (PHS): evaluation of radiation therapy long-term results and literature review. Radiotherapy and oncology : journal of the European Society for Therapeutic Radiology and Oncology. 1998;47(1):17-28.

121. Rubenthaler F, Wiese M, Senge A, Keller L, Wittenberg RH. Long-term follow-up of open and endoscopic Hohmann procedures for lateral epicondylitis. Arthroscopy : the journal of arthroscopic & related surgery : official publication of the Arthroscopy Association of North America and the International Arthroscopy Association. 2005;21(6):684-90.

122. Nirschl R. Elbow tendinosis/tennis elbow. Clinics in sports medicine. 1992;11(4):851-70.

123. Baskurt F, Ozcan A, Algun C. Comparison of effects of phonophoresis and iontophoresis of naproxen in the treatment of lateral epicondylitis. Clinical rehabilitation. 2003;17(1):96-100.

124. Connell DA, Ali KE, Ahmad M, Lambert S, Corbett S, Curtis M. Ultrasound-guided autologous blood injection for tennis elbow. Skeletal radiology. 2006;35(6):371-7.

125. Weber T, Kabelka B. Noninvasive monopolar capacitive-coupled radiofrequency for the treatment of pain associated with lateral elbow tendinopathies: 1-year follow-up. PM R. 2012;4(3):176-81.

126. Stenhouse G, Sookur P, Watson M. Do blood growth factors offer additional benefit in refractory lateral epicondylitis? A prospective, randomized pilot trial of dry needling as a stand-alone procedure versus dry needling and autologous conditioned plasma. Skeletal radiology. 2013;42(11):1515-20.

127. Organ SW, Nirschl RP, Kraushaar BS, Guidi EJ. Salvage surgery for lateral tennis elbow. The American journal of sports medicine. 1997;25(6):746-50.

128. Edwards SG, Calandruccio JH. Autologous blood injections for refractory lateral epicondylitis. The Journal of hand surgery. 2003;28(2):272-8.

129. Jindal N, Gaury Y, Banshiwal RC, Lamoria R, Bachhal V. Comparison of short term results of single injection of autologous blood and steroid injection in tennis elbow: a prospective study. J Orthop Surg Res. 2013;8(1):10.

130. Nirschl RP, Rodin DM, Ochiai DH, Maartmann-Moe C, Group D-A-S. Iontophoretic administration of dexamethasone sodium phosphate for acute epicondylitis. A randomized, double-blinded, placebo-controlled study. The American journal of sports medicine. 2003;31(2):189-95.

131. Kazemi M, Azma K, Tavana B, Rezaiee Moghaddam F, Panahi A. Autologous blood versus corticosteroid local injection in the short-term treatment of lateral elbow tendinopathy: a randomized clinical trial of efficacy. Am J Phys Med Rehabil. 2010;89(8):660-7.

132. Cho BK, Kim YM, Kim DS, Choi ES, Shon HC, Park KJ, et al. Mini-open muscle resection procedure under local anesthesia for lateral and medial epicondylitis. Clin Orthop Surg. 2009;1(3):123-7.

133. Dilek B, Batmaz I, Sariyildiz MA, Sahin E, Ilter L, Gulbahar S, et al. Kinesio taping in patients with lateral epicondylitis. J Back Musculoskeletal Rehabil. 2016;29(4):853-8.

134. Soeur L, Desmoineaux P, Devillier A, Pujol N, Beaufils P. Outcomes of arthroscopic lateral epicondylitis release: Should we treat earlier? Orthop Traumatol Surg Res. 2016;102(6):775-80.

135. Connell DA, Ali KE, Ahmad M, Lambert S, Corbett S, Curtis M. Ultrasound-guided autologous blood injection for tennis elbow. Skeletal radiology. 2006;35(6):371-7.

136. Stenhouse G, Sookur P, Watson M. Do blood growth factors offer additional benefit in refractory lateral epicondylitis? A prospective, randomized pilot trial of dry needling as a stand-alone procedure versus dry needling and autologous conditioned plasma. Skeletal radiology. 2013;42(11):1515-20.

137. Guyver PM, Cattell AE, Hall MJ, Brinsden MD. Oxford elbow scores in an asymptomatic population. Ann R Coll Surg Engl. 2013;95(6):415-7.

138. de Haan J, Schep N, Tuinebreijer W, Patka P, den Hartog D. Rasch analysis of the Dutch version of the Oxford elbow score. Patient Relat Outcome Meas. 2011;2:145-9.

139. de Haan J, Goei H, Schep NW, Tuinebreijer WE, Patka P, den Hartog D. The reliability, validity and responsiveness of the Dutch version of the Oxford elbow score. J Orthop Surg Res. 2011;6:39.

140. Ebrahimzadeh MH, Kachooei AR, Vahedi E, Moradi A, Mashayekhi Z, Hallaj-Moghaddam M, et al. Validity and cross-cultural adaptation of the persian version of the oxford elbow score. Int J Rheumatol. 2014;2014:381237.

141. Plaschke HC, Jørgensen A, Thillemann TM, Brorson S, Olsen BS. Validation of the Danish version of the Oxford elbow score. Danish Medical Journal. 2013;60(10).

142. Marquardt J, Schottker-Koniger T, Schafer A. [Validation of the German version of the Oxford Elbow Score : A cross-sectional study]. Orthopade. 2016;45(8):695-700.

143. Yosmaoglu HB, Dogan D, Sonmezer E. The reliability and validity of the Turkish version of the Oxford Elbow Score. Journal of Orthopaedic Surgery. 2016;11(1):95.

144. Tahririan MA, Moayednia A, Momeni A, Yousefi A, Vahdatpour B. A randomized clinical trial on comparison of corticosteroid injection with or without splinting versus saline injection with or without splinting in patients with lateral epicondylitis. Journal of Research in Medical Sciences. 2014;19(9):813-8.

145. Nazar M, Lipscombe S, Morapudi S, Tuvo G, Kebrle R, Marlow W, et al. Percutaneous tennis elbow release under local anaesthesia. Open Orthop J. 2012;6:129-32.

146. Carulli C, Tonelli F, Innocenti M, Gambardella B, Muncibi F, Innocenti M. Effectiveness of extracorporeal shockwave therapy in three major tendon diseases. J. 2016;17(1):15-20.

147. Chung B, Wiley JP. Validity, responsiveness and reliability of the Patient-Rated Tennis Elbow Evaluation. Hand Therapy. 2010;15(3):62-8 7p.

148. Overend TJ, Wuori-Fearn JL, Kramer JF, MacDermid JC. Reliability of a patient-rated forearm evaluation questionnaire for patients with lateral epicondylitis. Journal of Hand Therapy. 1999;12(1):31-7.

149. Poltawski L, Watson T. Measuring clinically important change with the Patient-rated Tennis Elbow Evaluation. Hand Therapy. 2011;16(3):52-7 6p.

150. Stratford P, Levy DR, Gauldie S, Levy K, Miseferi D. Extensor carpi radialis tendonitis: a validation of selected outcome measures. Physiotherapy Canada. 1987;39(4):250-5 6p.

151. Vincent J, MacDermid JC. Patient-rated tennis elbow evaluation questionnaire. J Physiother. 2014;60(4):240.

152. Altan L, Ercan I, Konur S. Reliability and validity of Turkish version of the patient rated tennis elbow evaluation. Rheumatol Int. 2010;30(8):1049-54.

153. Blanchette MA, Normand MC. Cross-cultural adaptation of the patient-rated tennis elbow evaluation to Canadian French. J Hand Ther. 2010;23(3):290-9; quiz 300.

154. Cacchio A, Necozione S, MacDermid JC, Rompe JD, Maffulli N, di Orio F, et al. Cross-cultural adaptation and measurement properties of the italian version of the Patient-Rated Tennis Elbow Evaluation (PRTEE) questionnaire. Phys Ther. 2012;92(8):1036-45.

155. Leung HB, Yen CH, Tse PYT. Reliability of Hong Kong Chinese version of the Patient-rated Forearm Evaluation Questionnaire for lateral epicondylitis. Hong Kong medical journal = Xianggang yi xue za zhi / Hong Kong Academy of Medicine. 2004;10(3):172-7.

156. Kaux JF, Delvaux F, Schaus J, Demoulin C, Locquet M, Buckinx F, et al. Cross-cultural adaptation and validation of the Patient-Rated Tennis Elbow Evaluation Questionnaire on lateral elbow tendinopathy for French-speaking patients. J Hand Ther. 2016;29(4):496-504.

157. Farazdaghi MR, Mansoori A, Vosoughi O, Kordi Yoosefinejad A. Evaluation of the reliability and validity of the Persian version of Patient-Rated Elbow Evaluation questionnaire. Rheumatol Int. 2017;37(5):743-50.

158. Connell D, Datir A, Alyas F, Curtis M. Treatment of lateral epicondylitis using skin-derived tenocyte-like cells. British journal of sports medicine. 2009;43(4):293-8.

159. Paoloni JA, Murrell GA, Burch RM, Ang RY. Randomised, double-blind, placebo-controlled clinical trial of a new topical glyceryl trinitrate patch for chronic lateral epicondylosis. Br J Sports Med. 2009;43(4):299-302.

160. Radpasand M, Owens E. Combined multimodal therapies for chronic tennis elbow: pilot study to test protocols for a randomized clinical trial. J Manipulative Physiol Ther. 2009;32(7):571-85.

161. Tarhan S, Unlu Z, Ovali GY, Pabuscu Y. Value of Ultrasonography on Diagnosis and Assessment of Pain and Grip Strength in Patients with Lateral Epicondylitis. Turk J Rheumatol. 2009;24(3):123-30.

162. Amro A, Diener I, Bdair WO, Hameda IM, Shalabi AI, Ilyyan DI. The effects of Mulligan mobilisation with movement and taping techniques on pain, grip strength, and function in patients with lateral epicondylitis. Hong Kong Physiotherapy Journal. 2010;28(1):19-23.

163. Clarke AW, Ahmad M, Curtis M, Connell DA. Lateral Elbow Tendinopathy Correlation of Ultrasound Findings With Pain and Functional Disability. The American journal of sports medicine. 2010;38(6):1209-14.

164. Blanchette MA, Normand MC. Augmented soft tissue mobilization vs natural history in the treatment of lateral epicondylitis: a pilot study. J Manipulative Physiol Ther. 2011;34(2):123-30.

165. Blanchette M-A, Normand MC. Impairment assessment of lateral epicondylitis through electromyography and dynamometry. Journal of the Canadian Chiropractic Association. 2011;55(2):96-106 11p.

166. Creaney L, Wallace A, Curtis M, Connell D. Growth factor-based therapies provide additional benefit beyond physical therapy in resistant elbow tendinopathy: a prospective, single-blind, randomised trial of autologous blood injections versus platelet-rich plasma injections. British journal of sports medicine. 2011:bjsports82503.

167. Gonzalez-Iglesias J, Cleland JA, del Rosario Gutierrez-Vega M, Fernandez-de-las-Penas C. Multimodal management of lateral epicondylalgia in rock climbers: a prospective case series. J Manipulative Physiol Ther. 2011;34(9):635-42.

168. Ajimsha MS, Chithra S, Thulasyammal RP. Effectiveness of myofascial release in the management of lateral epicondylitis in computer professionals. Arch Phys Med Rehabil. 2012;93(4):604-9.

169. Chourasia AO, Buhr KA, Rabago DP, Kijowski R, Irwin CB, Sesto ME. Effect of lateral epicondylosis on grip force development. J Hand Ther. 2012;25(1):27-36; quiz 7.

170. Forogh B, Khalighi M, Javanshir MA, Ghoseiri K, Kamali M, Raissi G. The effects of a new designed forearm orthosis in treatment of lateral epicondylitis. Disabil Rehabil Assist Technol. 2012;7(4):336-9.

171. Ilieva EM, Minchev RM, Petrova NS. Radial shock wave therapy in patients with lateral epicondylitis. Folia Med (Plovdiv). 2012;54(3):35-41.

172. Kim LJ, Choi H, Moon D. Improvement of Pain and Functional Activities in Patients with Lateral Epicondylitis of the Elbow by Mobilization with Movement: a Randomized, Placebo-Controlled Pilot Study. J Phys Ther Sci. 2012;24(9):787-90.

173. Chourasia AO, Buhr KA, Rabago DP, Kijowski R, Lee KS, Ryan MP, et al. Relationships Between Biomechanics, Tendon Pathology, and Function in Individuals With Lateral Epicondylosis. J Orthop Sport Phys. 2013;43(6):368-78.

174. Krogh TP, Fredberg U, Stengaard-Pedersen K, Christensen R, Jensen P, Ellingsen T. Treatment of lateral epicondylitis with platelet-rich plasma, glucocorticoid, or saline: a randomized, double-blind, placebo-controlled trial. Am J Sports Med. 2013;41(3):625-35.

175. Rabago D, Lee KS, Ryan M, Chourasia AO, Sesto ME, Zgierska A, et al. Hypertonic dextrose and morrhuate sodium injections (prolotherapy) for lateral epicondylosis (tennis elbow): results of a single-blind, pilot-level, randomized controlled trial. Am J Phys Med Rehabil. 2013;92(7):587-96.

176. Shin KM, Kim JH, Lee S, Shin MS, Kim TH, Park HJ, et al. Acupuncture for lateral epicondylitis (tennis elbow): study protocol for a randomized, practitioner-assessor blinded, controlled pilot clinical trial. Trials. 2013;14:174.

177. Shiple BJ. How effective are injection treatments for lateral epicondylitis? Clinical journal of sport medicine : official journal of the Canadian Academy of Sport Medicine. 2013;23(6):502-3.

178. Arik HO, Kose O, Guler F, Deniz G, Egerci OF, Ucar M. Injection of autologous blood versus corticosteroid for lateral epicondylitis: a randomised controlled study. J Orthop Surg (Hong Kong). 2014;22(3):333-7.

179. Lee S, Ko Y, Lee W. Changes in pain, dysfunction, and grip strength of patients with acute lateral epicondylitis caused by frequency of physical therapy: a randomized controlled trial. J Phys Ther Sci. 2014;26(7):1037-40.

180. Singh A, Gangwar DS, Singh S. Bone marrow injection: A novel treatment for tennis elbow. J Nat Sci Biol Med. 2014;5(2):389-91.

181. Coombes BK, Bisset L, Vicenzino B. Cold hyperalgesia associated with poorer prognosis in lateral epicondylalgia: a 1-year prognostic study of physical and psychological factors. Clin J Pain. 2015;31(1):30-5.

182. Murtezani A, Ibraimi Z, Vllasolli TO, Sllamniku S, Krasniqi S, Vokrri L. Exercise and Therapeutic Ultrasound Compared with Corticosteroid Injection for Chronic Lateral Epicondylitis: A Randomized Controlled Trial. Ortop Traumatol Rehabil. 2015;17(4):351-7.

183. Tosun HB, Gumustas S, Agir I, Uludag A, Serbest S, Pepele D, et al. Comparison of the effects of sodium hyaluronate-chondroitin sulphate and corticosteroid in the treatment of lateral epicondylitis: a prospective randomized trial. J Orthop Sci. 2015;20(5):837-43.

184. Qi L, Zhang Y-D, Yu R-B, Shi H-B. Magnetic Resonance Imaging of Patients With Chronic Lateral Epicondylitis: Is There a Relationship Between Magnetic Resonance Imaging Abnormalities of the Common Extensor Tendon and the Patient's Clinical Symptom? Medicine. 2016;95(5):1-5 p.

185. Faes M, van den Akker B, de Lint JA, Kooloos JGM, Hopman MTE. Dynamic extensor brace for lateral epicondylitis. Clinical orthopaedics and related research. 2006;442:149-57.

186. Nilsson P, Thom E, Baigi A, Marklund B, Mansson J. A prospective pilot study of a multidisciplinary home training programme for lateral epicondylitis. Musculoskeletal Care. 2007;5(1):36-50.

187. Bergin MJG, Hirata R, Mista C, Christensen SW, Tucker K, Vicenzino B, et al. Movement evoked pain and mechanical hyperalgesia after intramuscular injection of nerve growth factor: A model of sustained elbow pain. Pain Medicine. 2015;16(11):2180-91.

188. Coombes BK, Bisset L, Vicenzino B. Thermal hyperalgesia distinguishes those with severe pain and disability in unilateral lateral epicondylalgia. The Clinical journal of pain. 2012;28(7):595-601.

189. Dabholkar AS, Kalbande VM, Yardi S. Neural Tissue Mobilisation Using ULTT2b and Radial Head Mobilisation v/s Exercise Programme in Lateral Epicondylitis. Indian Journal of Physiotherapy and Occupational Therapy. 2013;7(4):247.

190. Maffulli G, Hemmings S, Maffulli N. Assessment of the effectiveness of extracorporeal shock wave therapy (ESWT) for soft tissue injuries (assert): an online database protocol. Translational medicine@ UniSa. 2014;10:46.

191. Massy-Westropp N, Simmonds S, Caragianis S, Potter A. Autologous blood injection and wrist immobilisation for chronic lateral epicondylitis. Advances in orthopedics. 2012;2012.

192. Tonks JH, Pai SK, Murali SR. Steroid injection therapy is the best conservative treatment for lateral epicondylitis: a prospective randomised controlled trial. International Journal of Clinical Practice. 2007;61(2):240-6.

193. Alizadehkhaiyat O, Fisher AC, Kemp GJ, Frostick SP. Pain, functional disability, and psychologic status in tennis elbow. The Clinical journal of pain. 2007;23(6):482-9.

194. Coombes BK, Connelly L, Bisset L, Vicenzino B. Economic evaluation favours physiotherapy but not corticosteroid injection as a first-line intervention for chronic lateral epicondylalgia: evidence from a randomised clinical trial. BJSM online. 2016;50(22):1400-5.

195. Segretin F, Paris G, Cheriet S, Delarue Y. Rehabilitation and auto-exercises protocol in patients with chronic lateral epicondylitis: 6 months follow-up. Ann Phys Rehabil Med. 2016;59S:e109.

196. Amroodi MN, Mahmuudi A, Salariyeh M, Amiri A. Surgical Treatment of Tennis Elbow; Minimal Incision Technique. Arch. 2016;4(4):366-70.

197. Merolla G, Dellabiancia F, Ricci A, Mussoni MP, Nucci S, Zanoli G, et al. Arthroscopic Debridement Versus Platelet-Rich Plasma Injection: A Prospective, Randomized, Comparative Study of Chronic Lateral Epicondylitis With a Nearly 2-Year Follow-Up. Arthroscopy. 2017;19:19.

198. Guo YH, Kuan TS, Chen KL, Lien WC, Hsieh PC, Hsieh IC, et al. Comparison Between Steroid and 2 Different Sites of Botulinum Toxin Injection in the Treatment of Lateral Epicondylalgia: A Randomized, Double-Blind, Active Drug-Controlled Pilot Study. Arch Phys Med Rehabil. 2017;98(1):36-42.

199. Capan N, Esmaeilzadeh S, Oral A, Basoglu C, Karan A, Sindel D. Radial Extracorporeal Shock Wave Therapy Is Not More Effective Than Placebo in the Management of Lateral Epicondylitis: A Double-Blind, Randomized, Placebo-Controlled Trial. Am J Phys Med Rehabil. 2016;95(7):495-506.

200. Tong C, Bertouch JVJV, Katrib A, Joshua F. Power Doppler ultrasound in lateral epicondylitis. Internal Medicine Journal. 2009;39:A40.

201. Clarke AW, Ahmad M, Curtis M, Connell DA. Lateral elbow tendinopathy: correlation of ultrasound findings with pain and functional disability. Am J Sports Med. 2010;38(6):1209-14.

202. Creaney L, Wallace A, Curtis M, Connell D. Growth factor-based therapies provide additional benefit beyond physical therapy in resistant elbow tendinopathy: a prospective, single-blind, randomised trial of autologous blood injections versus platelet-rich plasma injections. Br J Sports Med. 2011;45(12):966-71.

203. Beaton DE, Wright JG, Katz JN, Upper Extremity Collaborative G. Development of the QuickDASH: comparison of three item-reduction approaches. J Bone Joint Surg Am. 2005;87(5):1038-46.

204. Fan ZJ, Smith CK, Silverstein BA. Assessing validity of the QuickDASH and SF-12 as surveillance tools among workers with neck or upper extremity musculoskeletal disorders. Journal of Hand Therapy. 2008;21(4):354-65.

205. Polson K, Reid D, McNair PJ, Larmer P. Responsiveness, minimal importance difference and minimal detectable change scores of the shortened disability arm shoulder hand (QuickDASH) questionnaire. Manual therapy. 2010;15(4):404-7.

206. Smith-Forbes EV, Howell DM, Willoughby J, Pitts DG, Uhl TL. Specificity of the minimal clinically important difference of the quick Disabilities of the Arm Shoulder and Hand (QDASH) for distal upper extremity conditions. J Hand Ther. 2016;29(1):81-8.

207. Stover B, Silverstein B, Wickizer T, Martin DP, Kaufman J. Accuracy of a disability instrument to identify workers likely to develop upper extremity musculoskeletal disorders. Journal of occupational rehabilitation. 2007;17(2):227-45.

208. Solheim E, Hegna J, Oyen J. Extensor tendon release in tennis elbow: results and prognostic factors in 80 elbows. Knee Surg Sports Traumatol Arthrosc. 2011;19(6):1023-7.

209. Mardani-Kivi M, Karimi-Mobarakeh M, Karimi A, Akhoondzadeh N, Saheb-Ekhtiari K, Hashemi-Motlagh K, et al. The effects of corticosteroid injection versus local anesthetic injection in the treatment of lateral epicondylitis: a randomized single-blinded clinical trial. Arch Orthop Trauma Surg. 2013;133(6):757-63.

210. Solheim E, Hegna J, Oyen J. Arthroscopic versus open tennis elbow release: 3- to 6-year results of a case-control series of 305 elbows. Arthroscopy. 2013;29(5):854-9.

211. Wang A, Breidahl W, Mackie KE, Lin Z, Qin A, Chen J, et al. Autologous tenocyte injection for the treatment of severe, chronic resistant lateral epicondylitis: a pilot study. Am J Sports Med. 2013;41(12):2925-32.

212. Otoshi K, Takegami M, Sekiguchi M, Onishi Y, Yamazaki S, Otani K, et al. Chronic hyperglycemia increases the risk of lateral epicondylitis: the Locomotive Syndrome and Health Outcome in Aizu Cohort Study (LOHAS). Springerplus. 2015;4:407.

213. Trentini R, Mangano T, Repetto I, Cerruti P, Kuqi E, Trompetto C, et al. Short- to mid-term follow-up effectiveness of US-guided focal extracorporeal shock wave therapy in the treatment of elbow lateral epicondylitis. Musculoskelet Surg. 2015;99 Suppl 1:S91-7.

214. Wang A, Mackie K, Breidahl W, Wang T, Zheng MH. Evidence for the Durability of Autologous Tenocyte Injection for Treatment of Chronic Resistant Lateral Epicondylitis: Mean 4.5-Year Clinical Follow-up. Am J Sports Med. 2015;43(7):1775-83.

215. Kocyigit F, Kuyucu E, Kocyigit A, Herek DT, Savkin R, Aslan UB, et al. Association of real-time sonoelastography findings with clinical parameters in lateral epicondylitis. Rheumatol Int. 2016;36(1):91-100.

216. Walton MJ, Mackie K, Fallon M, Butler R, Breidahl W, Zheng MH, et al. The reliability and validity of magnetic resonance imaging in the assessment of chronic lateral epicondylitis. The Journal of hand surgery. 2011;36(3):475-9.

217. Glanzmann MC, Audige L. Platelet-rich plasma for chronic lateral epicondylitis: is one injection sufficient? Arch Orthop Trauma Surg. 2015;135(12):1637-45.

218. Solheim E, Hegna J, Oyen J, Inderhaug E. Arthroscopic Treatment of Lateral Epicondylitis: Tenotomy Versus Debridement. Arthroscopy. 2016;32(4):578-85.

219. Shillito M, Soong M, Martin N. Radiographic and Clinical Analysis of Lateral Epicondylitis. J Hand Surg [Am]. 2017;2017:12.

220. Kwon BC, Kim JY, Park KT. The Nirschl procedure versus arthroscopic extensor carpi radialis brevis debridement for lateral epicondylitis. J Shoulder Elbow Surg. 2017;26(1):118-24.

221. Gulabi D, Uysal MA, Akca A, Colak I, Cecen GS, Gumustas S. USG-guided injection of corticosteroid for lateral epicondylitis does not improve clinical outcomes: a prospective randomised study. Arch Orthop Trauma Surg. 2017;137(5):601-6.

222. Roles N, Maudsley R. RADIAL TUNNEL SYNDROME Resistant Tennis Elbow as a Nerve Entrapment. Journal of Bone & Joint Surgery, British Volume. 1972;54(3):499-508.

223. Perlick L, Gassel F, Zander D, Schmitt O, Wallny T. [Comparison of results of results of medium energy ESWT and Mittelmeier surgical therapy in therapy refractory epicondylitis humeri radialis]. Vergleich der Ergebnisse der mittelenergetischen ESWT und der operativen Therapie in der Technik nach Mittelmeier bei der therapieresistenten Epicondylitis humeri radialis. 1999;137(4):316-21.

224. Rompe JD, Riedel C, Betz U, Fink C. Chronic lateral epicondylitis of the elbow: a prospective study of low-energy shockwave therapy and low-energy shockwave therapy plus manual therapy of the cervical spine. Archives of Physical Medicine & Rehabilitation. 2001;82(5):578-82.

225. Haake M, Konig IR, Decker T, Riedel C, Buch M, Muller HH, et al. Extracorporeal shock wave therapy in the treatment of lateral epicondylitis : a randomized multicenter trial. The Journal of bone and joint surgery American volume. 2002;84-A(11):1982-91.

226. Pannier S, Masquelet AC. [Treatment of epicondylitis by deep fasciotomy of the extensor carpi radialis brevis and supinator: a review of 18 cases]. Traitement de l'epicondylalgie par aponevrotomie profonde de l'extensor carpi radialis brevis et du supinator: une serie de 18 cas. 2002;88(6):565-72.

227. Furia JP. Safety and efficacy of extracorporeal shock wave therapy for chronic lateral epicondylitis. American journal of orthopedics (Belle Mead, NJ). 2005;34(1):13-9.

228. Decker T, Kuhne B, Gobel F. [Extracorporeal shockwave therapy (ESWT) in epicondylitis humeri radialis. Short-term and intermediate-term results]. Extrakorporale Stosswellentherapie (ESWT) bei Epicondylitis humeri radialis Kurz- und mittelfristige Ergebnisse. 2002;31(7):633-6.

229. Vulpiani MC, Nusca SM, Vetrano M, Ovidi S, Baldini R, Piermattei C, et al. Extracorporeal shock wave therapy vs cryoultrasound therapy in the treatment of chronic lateral epicondylitis. One year follow up study. Muscles Ligaments Tendons J. 2015;5(3):167-74.

230. Haake M, Jensen K, Prinz H, Willenberg T. [Design of a multicenter study for assessing the effectiveness of extracorporeal shockwave therapy in epicondylitis humeri radialis]. Design einer Multizenterstudie zum Wirksamkeitsnachweis der Extrakorporalen Stosswellentherapie (ESTW) bei Epicondylitis humeri radialis. 2000;138(2):99-103.

231. Ozkut AT, Kilincoglu V, Ozkan NK, Eren A, Ertas M. [Extracorporeal shock wave therapy in patients with lateral epicondylitis]. Acta Orthop Traumatol Turc. 2007;41(3):207-10.

232. Wang AW, Erak S. Fractional lengthening of forearm extensors for resistant lateral epicondylitis. ANZ J Surg. 2007;77(11):981-4.

233. Montalvan B, Le Goux P, Klouche S, Borgel D, Hardy P, Breban M. Inefficacy of ultrasound-guided local injections of autologous conditioned plasma for recent epicondylitis: results of a double-blind placebo-controlled randomized clinical trial with one-year follow-up. Rheumatology (Oxford). 2016;55(2):279-85.

234. Lowe A, Wessel J, Battié MC. Test-retest reliability, construct validity, and responsiveness of a functional pain scale for tennis elbow. Physiotherapy Canada. 2003;55(2):114-22 9p.

235. Nagrale AV, Herd CR, Ganvir S, Ramteke G. Cyriax physiotherapy versus phonophoresis with supervised exercise in subjects with lateral epicondylalgia: a randomized clinical trial. J Man Manip Ther. 2009;17(3):171-8.

236. Viswas R, Ramachandran R, Korde Anantkumar P. Comparison of effectiveness of supervised exercise program and Cyriax physiotherapy in patients with tennis elbow (lateral epicondylitis): a randomized clinical trial. ScientificWorldJournal. 2012;2012:939645.

237. Pransky G, Feuerstein M, Himmelstein J, Katz JN, Vickers-Lahti M. Measuring functional outcomes in work-related upper extremity disorders: Development and validation of the upper extremity function scale. Journal of Occupational and Environmental Medicine. 1997;39(12):1195-202.

238. Rompe JD, Decking J, Schoellner C, Theis C. Repetitive low-energy shock wave treatment for chronic lateral epicondylitis in tennis players. American Journal of Sports Medicine. 2004;32(3):734-43 10p.

239. Pettrone FA, McCall BR. Extracorporeal shock wave therapy without local anesthesia for chronic lateral epicondylitis. Journal of Bone & Joint Surgery, American Volume. 2005;87-A(6):1297-304 8p.

240. Ozturan KE, Yucel I, Cakici H, Guven M, Sungur I. Autologous blood and corticosteroid injection and extracoporeal shock wave therapy in the treatment of lateral epicondylitis. Orthopedics. 2010;33(2):84-91.

241. Verhaar J, Walenkamp G, Kester A, Van Mameren H, Van der Linden T. Lateral extensor release for tennis elbow. A prospective long-term follow-up study. J Bone Joint Surg Am. 1993;75(7):1034-43.

242. Svernlov B, Adolfsson L. Non-operative treatment regime including eccentric training for lateral humeral epicondylalgia. Scandinavian journal of medicine & science in sports. 2001;11(6):328-34.

243. Svernlov B, Adolfsson L. Outcome of release of the lateral extensor muscle origin for epicondylitis. Scand J Plast Reconstr Surg Hand Surg. 2006;40(3):161-5.

244. Krischek O, Pompe JD, Hopf C, Vogel J, Herbsthofer B, Nafe B, et al. [Extracorporeal shockwave therapy in epicondylitis humeri ulnaris or radialis--a prospective, controlled, comparative study]. Die extrakorporale Stosswellentherapie bei Epicondylitis humeri ulnaris oder radialis--Eine prospektive, kontrollierte, vergleichende Studie. 1998;136(1):3-7.

245. Isikan UE, Sarban S, Kocabey Y. [The results of open surgical treatment in patients with chronic refractory lateral epicondylitis]. Kronik, konservatif tedaviye direncli lateral epikondilitte acik cerrahi tedavi sonuclari. 2005;39(2):128-32.

246. Ozden R, Uruc V, Doğramaci Y, Kalaci A, Yengil E. Management of tennis elbow with topical glyceryl trinitrate. Acta Orthop Traumato. 2013;48(2):175-80.
