# Supplementary material for: Assessing Patient-Centred Outcomes in Lateral Elbow Tendinopathy: A Systematic Review and Standardised Comparison of English Language Clinical Rating Systems
Source: Sports Med Open. 2019 Mar 20;5:10. doi: 10.1186/s40798-019-0183-2 (PMC6426924; doi:10.1186/s40798-019-0183-2)
Supplement: Supplementary file 3 — EMPRO attribute and individual item scores for each outcome instrument. Item scores graded from 4 (strongly agree) to 1 (strongly disagree or no information). (DOCX 33 kb) [file 40798_2019_183_MOESM3_ESM.docx]

Additional file 3

EMPRO Attribute and individual item scores for each outcome instrument. Item scores graded from 4 (strongly agree) to 1 (strongly disagree or no information).

Key: A&C (Andrews and Carson) ASES-e (American Shoulder and Elbow Score-e) DASH (Disabilities of the Arm, Shoulder and Hand) HSS (Hospital for Special Surgery) LES (Liverpool Elbow Score) MEPS (Mayo Elbow Performance Score) Morrey Nirschl OES (Oxford Elbow Score) PRTEE (Patient-Rated Tennis Elbow Evaluation) (formally PRFE) qDASH (quick Disabilities of the Arm Shoulder and Hand) R&M (Roles and Maudsley) TEFS (Tennis Elbow Functional Score) ULFI (Upper Limb Functional Index).

| Attributes and Items | A&C | ASES-e | DASH | HSS | LES | MEPS | Morrey | Nirschl | OES | PRTEE | qDASH | R&M | TEFS | ULFI | Verhaar |
| --- | --- | --- | --- | --- | --- | --- | --- | --- | --- | --- | --- | --- | --- | --- | --- |
| CONCEPTUAL AND MEASUREMENT MODEL |  |  |  |  |  |  |  |  |  |  |  |  |  |  |  |
| Concept of measurement stated | 3 | 4 | 4 | 2 | 3 | 4 | 3 | 4 | 4 | 4 | 4 | 1 | 3 | 3 | 2 |
| Obtaining and combining items described | 1 | 3 | 4 | 1 | 4 | 1 | 1 | 1 | 4 | 1 | 3 | 1 | 2 | 3 | 1 |
| Rationality for dimensionality and scales | 1 | 2 | 3 | 1 | 1 | 1 | 1 | 1 | 3 | 2 | 4 | 1 | 1 | 2 | 1 |
| Involvement of the target population | 1 | 2 | 3 | 1 | 4 | 1 | 1 | 1 | 4 | 2 | 2 | 1 | 2 | 3 | 1 |
| Scale variability described and adequate | 1 | 1 | 3 | 2 | 2 | 2 | 2 | 2 | 3 | 3 | 3 | 1 | 3 | 3 | 1 |
| Level of measurement described | 2 | 1 | 3 | 2 | 3 | 2 | 2 | 1 | 3 | 1 | 3 | 1 | 2 | 3 | 1 |
| Procedures for deriving scores | 2 | 1 | 3 | 1 | 3 | 2 | 2 | 2 | 4 | 3 | 4 | 1 | 1 | 3 | 1 |
| ATTRIBUTE SCORE | 19.0 | 33.3 | 76.2 | 14.3 | 61.9 | 28.6 | 23.8 | 23.8 | 85.7 | 42.9 | 76.2 | 0.0 | 33.3 | 61.9 | 4.8 |
| RELIABILITY: Internal consistency |  |  |  |  |  |  |  |  |  |  |  |  |  |  |  |
| Data collection methods described | 1 | 1 | 3 | 1 | 3 | 1 | 1 | 1 | 4 | 3 | 3 | 1 | 1 | 3 | 1 |
| Cronbach's alpha adequate | 1 | 1 | 4 | 1 | 4 | 1 | 1 | 1 | 4 | 4 | 4 | 1 | 2 | 3 | 1 |
| IRT estimates provided | 1 | 1 | 1 | 1 | 1 | 1 | 1 | 1 | 1 | 1 | 1 | 1 | 1 | 1 | 1 |
| Testing in different populations | 1 | 1 | 2 | 1 | 3 | 1 | 1 | 1 | 2 | 4 | 3 | 1 | 1 | 2 | 1 |
| ATTRIBUTE SCORE | 0.0 | 0.0 | 50.0 | 0.0 | 58.3 | 0.0 | 0.0 | 0.0 | 58.3 | 66.7 | 58.3 | 0.0 | 8.3 | 41.7 | 0.0 |
| RELIABILITY: Reproducibility |  |  |  |  |  |  |  |  |  |  |  |  |  |  |  |
| Data collection methods described | 1 | 3 | 4 | 1 | 2 | 2 | 1 | 1 | 3 | 4 | 3 | 1 | 4 | 1 | 1 |
| Test-retest and time interval adequate | 1 | 1 | 4 | 1 | 2 | 2 | 1 | 1 | 2 | 3 | 3 | 1 | 4 | 1 | 1 |
| Reproducibility coefficients adequate | 1 | 4 | 4 | 1 | 2 | 1 | 1 | 1 | 4 | 4 | 4 | 1 | 4 | 1 | 1 |
| IRT estimates provided | 1 | 1 | 1 | 1 | 1 | 1 | 1 | 1 | 1 | 1 | 1 | 1 | 1 | 1 | 1 |
| ATTRIBUTE SCORE | 0.0 | 41.7 | 75.0 | 0.0 | 25.0 | 16.7 | 0.0 | 0.0 | 50.0 | 66.7 | 58.3 | 0.0 | 75.0 | 0.0 | 0.0 |
| VALIDITY |  |  |  |  |  |  |  |  |  |  |  |  |  |  |  |
| Content Validity adequate | 1 | 2 | 4 | 1 | 4 | 1 | 1 | 2 | 2 | 1 | 3 | 1 | 1 | 3 | 1 |
| Construct/critereon validity adequate | 2 | 3 | 3 | 3 | 3 | 4 | 3 | 1 | 4 | 3 | 4 | 2 | 4 | 3 | 1 |
| Sample composition described | 1 | 1 | 1 | 3 | 3 | 1 | 3 | 1 | 2 | 4 | 2 | 2 | 2 | 2 | 1 |
| Prior hypothesis stated | 1 | 4 | 3 | 1 | 1 | 4 | 1 | 1 | 4 | 4 | 3 | 1 | 2 | 2 | 1 |
| Rational for criterion validity | 1 | 1 | 1 | 1 | 1 | 1 | 1 | 1 | 1 | 1 | 4 | 1 | 1 | 1 | 1 |
| Tested in different populations | 1 | 1 | 3 | 1 | 3 | 1 | 1 |  | 1 |  | 3 |  |  | 2 |  |
| ATTRIBUTE SCORE | 5.6 | 33.3 | 50.0 | 22.2 | 50.0 | 33.3 | 22.2 | 6.7 | 44.4 | 53.3 | 72.2 | 13.3 | 33.3 | 38.9 | 0.0 |
| RESPONSIVENESS |  |  |  |  |  |  |  |  |  |  |  |  |  |  |  |
| Adequacy of methods | 2 | 1 | 4 | 1 | 2 | 4 | 1 | 1 | 4 | 4 | 4 | 2 | 4 | 4 | 1 |
| Description of estimated magnitude of change | 2 | 1 | 4 | 1 | 1 | 4 | 1 | 1 | 4 | 3 | 4 | 3 | 2 | 3 | 1 |
| Comparison of stable and unstable groups | 1 | 1 | 1 | 1 | 1 | 1 | 1 | 1 | 2 | 3 | 3 | 1 | 1 | 1 | 1 |
| ATTRIBUTE SCORE | 22.2 | 0.0 | 66.7 | 0.0 | 11.1 | 66.7 | 0.0 | 0.0 | 77.8 | 77.8 | 88.9 | 33.3 | 44.4 | 55.6 | 0.0 |
| INTERPRETABILITY |  |  |  |  |  |  |  |  |  |  |  |  |  |  |  |
| Rational of external criteria | 1 | 1 | 4 | 1 | 1 | 2 | 2 | 1 | 3 | 3 | 4 | 1 | 2 | 1 | 1 |
| Description of interpretation strategies | 1 | 1 | 3 | 1 | 1 | 1 | 1 | 1 | 3 | 3 | 4 | 1 | 2 | 1 | 1 |
| How data should be reported stated | 1 | 1 | 2 | 1 | 1 | 2 | 1 | 1 | 3 | 1 | 1 | 1 | 1 | 1 | 1 |
| ATTRIBUTE SCORE | 0.0 | 0.0 | 66.7 | 0.0 | 0.0 | 22.2 | 11.1 | 0.0 | 66.7 | 44.4 | 66.7 | 0.0 | 22.2 | 0.0 | 0.0 |
| BURDEN: Respondent |  |  |  |  |  |  |  |  |  |  |  |  |  |  |  |
| Skills and time needed | 1 | 1 | 3 | 2 | 3 | 1 | 2 | 1 | 2 | 2 | 2 | 1 | 1 | 2 | 1 |
| Impact on respondents | 1 | 1 | 4 | 1 | 2 | 1 | 1 | 1 | 3 | 3 | 3 | 1 | 1 | 1 | 1 |
| Not suitable circumstances | 1 | 1 | 4 | 1 | 1 | 1 | 1 | 1 | 2 | 3 | 3 | 1 | 1 | 1 | 1 |
| ATTRIBUTE SCORE | 0.0 | 0.0 | 88.9 | 11.1 | 33.3 | 0.0 | 11.1 | 0.0 | 44.4 | 55.6 | 55.6 | 0.0 | 0.0 | 11.1 | 0.0 |
| BURDEN: Administrative |  |  |  |  |  |  |  |  |  |  |  |  |  |  |  |
| Resources Required | 1 | 1 | 4 | 3 | 4 | 1 | 2 | 1 | 4 | 4 | 3 | 1 | 1 | 1 | 1 |
| Time required | 1 | 1 | 1 | 1 | 4 | 1 | 1 | 1 | 4 | 1 | 1 | 1 | 1 | 1 | 1 |
| Training and expertise needed | 1 | 1 | 1 | 1 | 1 | 1 | 1 | 1 | 4 | 1 | 2 | 1 | 1 | 1 | 1 |
| Burden of score calculation | 2 | 1 | 4 | 2 | 3 | 3 | 2 | 1 | 4 | 4 | 4 | 1 | 1 | 2 | 1 |
| ATTRIBUTE SCORE | 8.3 | 0.0 | 50.0 | 25.0 | 66.7 | 16.7 | 16.7 | 0.0 | 100.0 | 50.0 | 50.0 | 0.0 | 0.0 | 8.3 | 0.0 |
| OVERALL SCORE | **N/A** | **21.67** | **66.90** | **N/A** | **36.27** | **33.49** | **N/A** | **N/A** | **66.59** | **57.02** | **72.46** | **N/A** | **41.67** | **39.60** | **N/A** |
